# Transfer-Free Conformal Graphene Coating on Pyramidal Microstructures Decorated with Silver Nanoparticles for Superior Raman Signal Enhancement

**DOI:** 10.1021/acsami.5c11957

**Published:** 2025-09-12

**Authors:** Cheuk Yui Lai, Yu-Xuan Lin, En-Jing Lin, Ching-Chih Lin, Chiao-Chen Chen

**Affiliations:** Department of Chemistry, 34912 National Cheng Kung University, No.1, University Road, Tainan City 701, Taiwan

**Keywords:** pyramid-textured surface, conformal graphene coatings, transfer-free graphene, AgNPs/graphene hybrid system, surface-enhanced Raman scattering (SERS)

## Abstract

Graphene, a two-dimensional nanomaterial with excellent physicochemical properties, has considerable potential to functionalize surfaces for diverse applications. However, reliable methods for preparing uniform conformal graphene on complex surfaces are still limited. In this study, we develop a practical strategy for the direct growth of conformal graphene coatings on silicon substrates textured with randomly distributed micropyramidal structures. The produced transfer-free graphene exhibits high uniformity (monolayer content ∼95%), low defect density, and excellent conformality, even across high-curvature features, such as micropyramid apexes. The graphene films not only faithfully replicate the underlying microstructures but also contribute to advantageous surface properties, including strong fluorescence quenching, excellent chemical stability, enhanced molecular adsorption, and improved charge-transfer interactions. All these properties are crucial for effective surface-enhanced Raman scattering (SERS). This graphene-coated pyramidal substrate enabled reproducible and stable SERS detection of rhodamine 6G (R6G), exhibiting high sensitivity with a detection limit of ∼10^–6^ M, excellent long-term stability over 30 days, and low spatial signal variation of ∼10% at the millimeter scale. By further decorating the graphene-coated pyramidal substrate with silver nanoparticles, the detection limit was improved to 5.5 × 10^–9^ M for R6G with a high analytical enhancement factor of 1.08 × 10^5^. This enhanced performance arose from the synergistic interplay between the light-trapping capability of the microstructured surface, the chemical enhancement caused by the graphene interface, and the electromagnetic amplification provided by the plasmonic nanoparticles. These findings offer valuable insights into the design of high-performance SERS platforms. This study also presents a practical method for the direct synthesis of conformal graphene for surface functionalization that is promising for a wide range of applications in sensing, optoelectronics, and catalysis.

## Graphical abstract

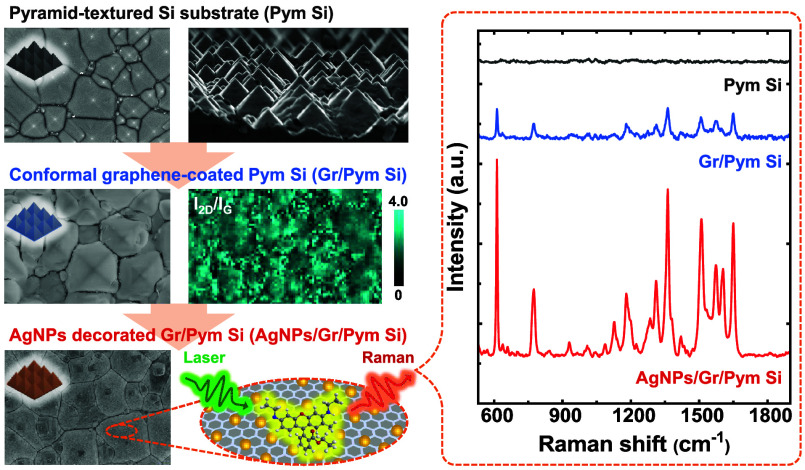

## 1 Introduction

Textured surfaces, particularly those with microscale or nanoscale features, play a critical role in devices for diverse applications, including optoelectronics, microfluidics, catalysis, and sensing.^1–4^ Complex geometries, such as pyramidal or pillar-like structures, can substantially enhance the light-trapping, fluid transport, molecular adsorption, and the specific surface area of textured surfaces, thereby improving the performance of optical, thermal, and sensing systems.^5,6^ However, to fully harness their potential, textured surfaces often require advanced modification with appropriate functional materials. Such surface modification not only enhances the intrinsic properties of textured surfaces but also leads to additional properties that cannot be achieved through topographical engineering alone, such as chemical stability, high electrical conductivity, and biocompatibility.^7,8^

Conventional surface modification techniques, including physical deposition and chemical grafting, are commonly employed to produce functional coatings.^9,10^ Although these methods are generally effective for planar or moderately rough surfaces, they have notable limitations when applied to substrates with high aspect ratios or sharp-edged microstructures. For example, physical deposition methods are often affected by shadowing effects, leading to incomplete or nonuniform coating, especially on sidewalls and recessed regions.^11^ Chemical grafting techniques are more adaptable to irregular surfaces than are physical deposition methods, but are often constrained by limited material compatibility and poor reproducibility.^12^ Consequently, achieving conformal, uniform, and chemically stable coatings with structural continuity over complex surface topographies remains challenging.

Graphene has emerged as an excellent material for surface functionalization because of its high mechanical flexibility, electrical conductivity, chemical stability, and optical transparency.^13–15^ Various approaches have been employed to coat surfaces with graphene, including the transfer of synthesized and exfoliated graphene films and the drop-casting of graphene oxide suspensions.^16–19^ However, these methods often result in graphene coatings with poor uniformity,^17,18^ insufficient adhesion,^17,18^ high defect density,^16,19^ and limited film continuity,^17–19^ particularly when they are applied to nonplanar surfaces with complex three-dimensional (3D) structures. The direct synthesis of graphene on target substrates offers a promising alternative to the aforementioned methods, eliminating the need for cumbersome transfer processes and enabling the formation of continuous, high-quality graphene films with improved uniformity and structural integrity.^20,21^ This method has potential for producing conformal graphene coatings on substrates with various geometries. However, studies on the direct growth of graphene on textured or nonplanar surfaces have reported several limitations, such as high defect density,^22–24^ limited conformity to only macroscale features,^25^ and the formation of vertically oriented nanosheets rather than smooth, continuous films.^16,23,24^ To the best of our knowledge, no reliable and scalable method has yet been proposed for synthesizing conformal graphene films with seamless adherence, low defect density, and uniform coverage over microscale 3D structures.

To address this research gap, we developed a feasible method for the direct synthesis of high-quality conformal graphene coatings on microscale 3D structures, employing pyramid-textured silicon substrates as a representative platform for graphene synthesis. These substrates, which are prepared through the anisotropic alkaline etching of single-crystal silicon, have excellent light-trapping capabilities and have been extensively used in photovoltaic and optoelectronic devices.^4,26^ Functionalizing such textured surfaces with directly grown graphene results in several advantageous properties relevant to surface-enhanced Raman scattering (SERS) applications, including effective fluorescence quenching,^27^ good chemical stability,^28^ enhanced molecular adsorption,^29^ and improved charge-transfer interactions with adsorbed species.^30–32^ The resulting graphene-coated pyramidal surfaces preserve their original 3D microstructural topography while providing a robust and active interface for sensitive SERS detection. To demonstrate the functional potential of these surfaces, we evaluated their SERS detection performance by using rhodamine 6G (R6G) as a model analyte. Further enhancement in SERS activity was achieved by decorating the conformal graphene layer with silver nanoparticles (AgNPs), resulting in a hybrid substrate capable of quantitative and reproducible detection of R6G at concentrations as low as 10^–8^ M with a high analytical enhancement factor (AEF) of 1.08 × 10^5^. The proposed method facilitates the fabrication of functional graphene-coated microstructured platforms, thereby opening new avenues for advanced SERS detection and other surface-based applications.

## 2 Experimental Section

### 2.1 Texturization of Silicon Substrates via Anisotropic Wet Etching

The substrates used for SERS detection in this study were fabricated on silicon wafers textured with pyramidal microstructures through a well-established anisotropic wet etching process. Alkaline etchants, such as potassium hydroxide (KOH), tetramethylammonium hydroxide, and ammonium hydroxide (NH_4_OH), are commonly employed in anisotropic wet etching.^33^ KOH was selected as the etchant in this study because of its excellent anisotropic etching selectivity, high efficiency, and relatively low chemical hazard. Monocrystalline silicon wafers (diameter: 2 in., orientation: ⟨100⟩, and thickness: 525 μm) were used as the starting material. Before etching, these wafers were immersed in preheated phosphoric acid (∼89%) at 165 °C for 15 min to remove the native silicon oxide layer.^34^ The wafers were then subjected to anisotropic etching in an aqueous solution containing 20 wt % KOH and 3 wt % isopropanol at 80 °C for 30 min (Figure 1). The etching solution was continuously stirred at a speed of 500 rpm in a temperature-controlled water bath to ensure uniform reaction conditions. Because of the anisotropic etching behavior of KOH, which preferentially removes silicon along the ⟨100⟩ direction, well-defined pyramidal microstructures were formed on the wafer surface. Surface microstructures with varied sizes and morphologies—including pyramids, octagonal pyramids, and cones—can be generated by adjusting the KOH concentration, reaction temperature, etching time, and specific additives. In this study, the etching parameters were optimized to fabricate uniformly distributed pyramidal microstructures. After etching, the substrates were ultrasonicated for 10 min and thoroughly rinsed with deionized (DI) water to remove residual contaminants, following which they were blow-dried with nitrogen.

**Figure 1:**
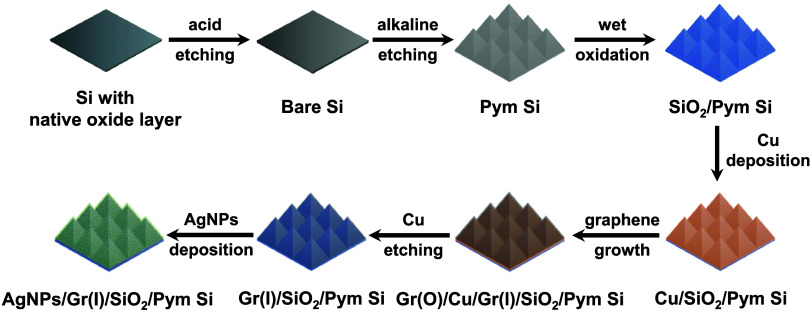
Fabrication process for pyramid-textured silicon substrates coated with conformal graphene and decorated with AgNPs. Flat silicon wafers were first textured to produce micropyramids on them (SiO_2_/Pym Si). Subsequently, Cu deposition was conducted to create catalytic substrates (Cu/SiO_2_/Pym Si) for the CVD synthesis of transfer-free graphene. During the CVD process, graphene layers formed on the upper Cu surface and at the Cu–SiO_2_ interface, with Gr­(O)/Cu/Gr­(I)/SiO_2_/Pym Si substrates being produced. Removal of the outer graphene layer and Cu film resulted in a conformal graphene coating on the pyramidal surface [Gr­(I)/SiO_2_/Pym Si]. Subsequent AgNP deposition resulted in the production of AgNPs/Gr­(I)/SiO_2_/Pym Si substrates, which served as SERS surfaces for the sensitive and reproducible detection of R6G.

### 2.2 Preparation of a Transfer-Free Conformal Graphene Coating on Pyramidal SiO_2_/Si Substrates

To prepare a suitable catalytic substrate for the synthesis of transfer-free graphene on textured substrates through chemical vapor deposition (CVD), a 300 nm-thick amorphous SiO_2_ buffer layer was thermally grown on the prepared pyramid-textured silicon wafers in a wet-oxidation furnace (SJ-CA1200-D4, SJ High Technology Company, Taiwan). Subsequently, a catalytic Cu film was deposited on each textured SiO_2_/Si substrate through ion-beam sputtering under an Ar atmosphere at a pressure of 7.6 × 10^–3^ Torr, with the initial chamber pressure being 4.0 × 10^–6^ Torr.^20,21^ The Cu film was deposited at a rate of 0.9 Å/s until it reached an optimal thickness of 950 nm. The SiO_2_ buffer layer is crucial for the formation of high-quality, transfer-free graphene by preventing the formation of copper oxides or Cu–Si alloy and minimizing the sublimation of Cu at elevated temperatures (>1000 °C).^35^

The resulting stack comprised a 950 nm-thick Cu layer on a 300 nm-thick SiO_2_-coated, pyramid-textured silicon substrate (denoted as Cu/SiO_2_/Pym Si in Figure 1). This stack was cut into pieces of 0.75 × 0.75 cm^2^ to serve as catalytic substrates for the CVD synthesis of transfer-free graphene through a spatial confinement approach adopted from our previous work.^20,21,36^ The synthesis process was conducted in a quartz slit reactor with a confined reaction space (85 × 13 × 0.55 mm^3^), with the Cu/SiO_2_/Pym Si substrate inserted into the chamber for the direct growth of graphene at the Cu–SiO_2_ interface. In contrast to our previous studies focusing on planar surfaces,^20,21^ this study prepared the substrate surface with pyramidal microstructures. The substrates produced following CVD synthesis consisted of graphene layers on the Cu upper surface and at the Cu–SiO_2_ interface. These substrates are denoted as Gr­(O)/Cu/Gr­(I)/SiO_2_/Pym Si (Figure 1). To expose the graphene layer grown at the Cu–SiO_2_ interface, the deposited Cu film and the graphene layer grown on it [Gr­(O)] were removed using a laminar flow-assisted etching process developed by our group.^21^ This well-controlled etching procedure preserved the integrity of the interfacial graphene [Gr­(I)], resulting in a transfer-free conformal graphene film uniformly coating the pyramid-textured SiO_2_/Si substrate [denoted as Gr­(I)/SiO_2_/Pym Si in Figure 1]. Details regarding the CVD parameters and etching procedures are provided in Sections 1 and 2 of the Supporting Information (Figures S1–S3).

The quality and uniformity of the transfer-free conformal graphene coatings were evaluated through micro-Raman spectroscopy (inVia Reflex, Renishaw, UK). This evaluation was conducted with a 532 nm solid-state laser (50 mW full-scale power) operated at 5 mW (10% of full power) with an exposure time of 1.0 s. The laser was focused through a 100× objective, producing a spot size of approximately 1 μm^2^. Two-dimensional Raman mapping was conducted over a 90 × 50 μm^2^ area with a spatial resolution of 90 × 50 pixels (1 μm/pixel), and an exposure time of 1.0 s per measurement point.

### 2.3 Fabrication of AgNPs/Graphene-Coated Pyramidal SiO_2_/Si Substrates for SERS Applications

The prepared Gr­(I)/SiO_2_/Pym Si substrates were decorated with AgNPs through electron-beam (e-beam) evaporation. AgNPs were deposited at a rate of 0.2 Å/s under an Ar atmosphere with a pressure of 5.0 × 10^–6^ Torr. To optimize AgNP coatings, deposition was conducted for 50, 100, 200, 250, and 300 s. The AgNP-decorated Gr­(I)/SiO_2_/Pym Si substrates [denoted as AgNPs/Gr­(I)/SiO_2_/Pym Si in Figure 1] were then employed as SERS substrates, using R6G (Sigma-Aldrich, ∼95%) as the model analyte to evaluate their detection performance. Raman measurements of R6G on AgNPs/Gr­(I)/SiO_2_/Pym Si and other substrates prepared in this study were conducted using a micro-Raman spectrometer (inVia Reflex, Renishaw, UK). Unless otherwise noted, all single-point Raman spectra and two-dimensional Raman maps of R6G were acquired using a 532 nm solid-state laser (50 mW full-scale power), operated at 0.25 mW (0.5% of full power), with an exposure time of 1.0 s per measurement point. The laser was focused through a 100× objective, producing a spot size of approximately 1 μm^2^. Details about other sample characterization methods, including scanning electron microscopy (SEM) and X-ray photoelectron spectroscopy (XPS) are described in Section 3 of the Supporting Information.

## 3 Results and Discussion

### 3.1 Fabrication and Characterization of Pyramidal SiO_2_/Si Substrates

Alkaline etching of silicon involves two fundamental steps: slow surface oxidation catalyzed by hydroxide (OH^–^) ions (eq 1), followed by the rapid dissolution of oxidized silicon in H_2_O (eq 2).^33^ In the initial oxidation step, OH^–^ ions react with the hydrogen-terminated Si surface, replacing Si–H bonds with Si–OH groups. This substitution, which is driven by the high electronegativity of oxygen, leads to the oxidation of surface Si atoms and the weakening of the underlying Si–Si backbones (eq 1). Subsequently, polar H_2_O molecules react with the weakened Si–Si network, leading to the formation of soluble orthosilicate acid [Si­(OH)_4_], which is dissolved from the substrate surface, resulting in the etching of the silicon substrate (eq 2). The generated Si­(OH)_4_ can decompose into metasilicic acid (H_2_SiO_3_) and H_2_O (eq 3), following which H_2_SiO_3_ is neutralized by OH^–^ ions to form soluble silicate ions (SiO_3_^2–^) (eq 4).^33,37^

1 $${\left(=Si\right)}_{2}SiH_{2}+2{OH}^{-}+2H_{2}O\to {\left(=Si\right)}_{2}Si{\left(OH\right)}_{2}+2H_{2}↑+2{OH}^{-}$$

2 $${\left(=\mathrm{Si}\right)}_{2}\mathrm{Si}{\left(\mathrm{OH}\right)}_{2}+2H_{2}O\to 2\left(=\mathrm{SiH}\right)+\mathrm{Si}{\left(\mathrm{OH}\right)}_{4}$$

3 $$\mathrm{Si}{\left(\mathrm{OH}\right)}_{4}\to H_{2}\mathrm{Si}O_{3}+H_{2}O$$

4 $$H_{2}\mathrm{Si}O_{3}+2{\mathrm{OH}}^{-}\to \mathrm{Si}O_{3}^{2-}+2H_{2}O$$

When ⟨100⟩-oriented Si wafers are used for alkaline etching, KOH etches silicon anisotropically, preferentially attacking the (100) planes. This process leads to the formation of pyramid-like surface features bounded by^38^ planes that undergo slow etching and display a characteristic sidewall angle of 54.7° (Figure 2a). Consequently, uniformly textured Si substrates with pyramidal microstructures are produced. By using the optimized etching protocol described in Section 2.1, we fabricated Si substrates with densely packed pyramidal structures exhibiting an average height of approximately 4.92 μm, as confirmed by top-view (Figure 2b) and cross-sectional (Figure 2c) scanning electron microscopy (SEM) images. The base size distribution of the pyramidal structures shown in Figure 2b was analyzed by ImageJ.^39^ This distribution was presented as a histogram in Figure 2d, with the average base length of approximately 6.55 μm.

**Figure 2:**
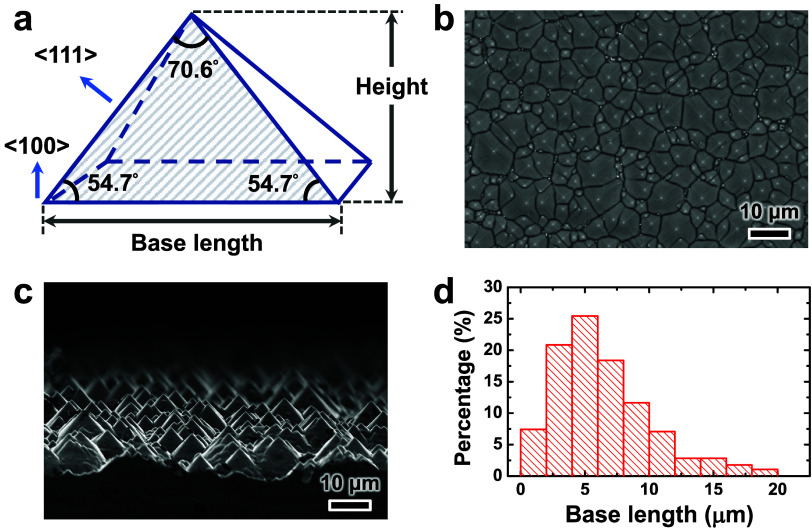
(a) Schematic of a typical square-base micropyramidal structure formed through the anisotropic wet etching of a monocrystalline Si substrate with a ⟨100⟩ orientation. The {111} facets and interior angles of this micropyramidal structure are shown in (a). (b) Top-view and (c) cross-sectional SEM images of micropyramids formed on textured silicon. (d) Histogram of the base lengths of the micropyramids in (b).

### 3.2 Preparation and Characterization of Conformal Graphene Monolayers on Pyramidal SiO_2_/Si Substrates

To enable the synthesis of transfer-free graphene on a textured substrate, the fabricated pyramidal silicon substrates (Pym Si) were first subjected to a wet-oxidation process to produce an amorphous SiO_2_ buffer layer (∼300 nm thick), resulting in SiO_2_/Pym Si substrates. SEM characterization (Figure 3a) confirmed that the formation of the SiO_2_ buffer layer did not distort the underlying pyramidal microstructure. Furthermore, high-magnification imaging (Figure 3d) revealed that the SiO_2_ surface was smooth and free of particulate contamination, highlighting the structural stability and cleanliness of the produced SiO_2_/Pym Si substrates. After the wet-oxidation process, a 950 nm-thick Cu film was deposited on each SiO_2_/Pym Si substrate to prepare Cu/SiO_2_/Pym Si catalytic substrates for the CVD synthesis of graphene (Figure 3b). The high-resolution SEM image in Figure 3e indicates that the deposited Cu film consisted of densely packed Cu nanoparticles, which resulted in a rougher surface than that of the bare SiO_2_ coating. Nevertheless, the pyramidal topography remained intact (Figure S4). The Cu-coated substrates were then employed for the CVD synthesis of graphene through a spatial confinement approach adopted from our previous study.^20^

**Figure 3:**
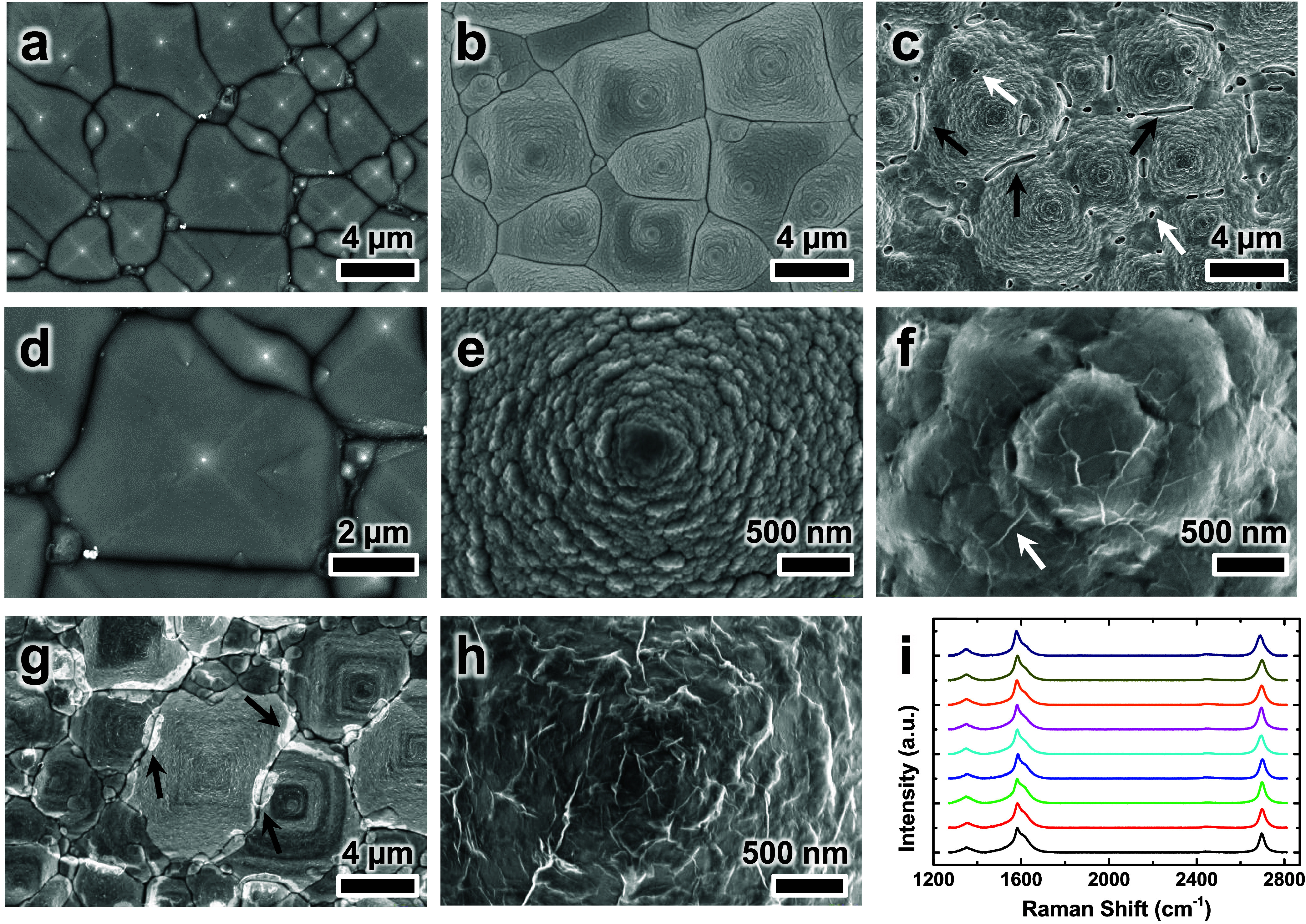
(a–h) Top-view SEM images of substrates produced in different fabrication stages, recorded at low (a–c, g) and high (d–f, h) magnifications: (a, d) SiO_2_/Pym Si, (b, e) Cu/SiO_2_/Pym Si, (c, f) Gr­(O)/Cu/Gr­(I)/SiO_2_/Pym Si, and (g, h) Gr­(O)/Gr­(I)/SiO_2_/Pym Si substrates. In (c), white and black arrows highlight pinholes and strip-like cracks, respectively, induced by Cu sublimation during the CVD process. In (f), a white arrow points to a characteristic graphene wrinkle on the upper Cu surface. In (g), black arrows indicate cracks in the produced graphene film. (i) Raman spectra collected at nine randomly selected locations on a representative Gr­(O)/Gr­(I)/SiO_2_/Pym Si substrate.

Following the CVD process, the morphological changes in the Cu layer were examined. Pinholes (Figure 3c, white arrows) and elongated cracks (Figure 3c, black arrows) appeared in the Cu film, particularly in the valleys between adjacent pyramids. These defects were attributed to Cu sublimation at an elevated synthesis temperature (950 °C). The high-magnification SEM image (Figure 3f) revealed substantial thermal aggregation of Cu nanoparticles, with particle diameters increasing from <100 nm (Figure 3e) to ∼1 μm (Figure 3f). Moreover, wrinkled thin films (Figure 3f, white arrow), which were absent before the CVD process (Figure 3e), were observed on the Cu surface. The wrinkles were indicative of the formation of graphene films [Gr­(O)] on the upper Cu surface. Raman spectra acquired from the upper Cu surface confirmed the existence of graphene, with characteristic peaks observed at ∼1585 cm^–1^ (G band) and ∼2696 cm^–1^ (2D band), although significant photoluminescence from the Cu film interfered with the Raman measurement (Figure S5).^40^ Graphene growth also occurred at the Cu–SiO_2_ interface, resulting in the formation of an interfacial graphene layer [Gr­(I)].^20^ Consequently, the samples obtained after the CVD process comprised graphene layers above and below the Cu film [denoted as Gr­(O)/Cu/Gr­(I)/SiO_2_/Pym Si in Figure 1].

To produce a conformal graphene monolayer coating on the pyramidal SiO_2_/Si surface, the Cu film was removed through a laminar flow-assisted etching process conducted in a microfluidic system.^21^ Before the laminar flow-assisted etching process, a critical pre-etching step was conducted outside the microfluidic chamber to remove Gr­(O). Specifically, the Gr­(O)/Cu/Gr­(I)/SiO_2_/Pym Si substrates were immersed in 0.05 M ammonium persulfate (APS) solution for 5 min, following which they were subjected to ultrasonication for 10 min and then thoroughly rinsed with DI water. This pre-etching treatment was essential for avoiding the deposition of Gr­(O) on the underlying Gr­(I)/SiO_2_/Pym Si substrate during laminar flow-assisted Cu etching. Without pre-etching, the Gr­(O) layer on the upper Cu surface would have remained stacked on the interfacial Gr­(I) layer, forming a multilayer graphene structure [Gr­(O)/Gr­(I)/SiO_2_/Pym Si]. This problem would arise because the laminar flow within the microfluidic etching chamber lacked sufficient turbulence to remove the conformal Gr­(O) layer from the pyramidal surface topography. To verify the necessity of the pre-etching process for achieving a truly monolayer conformal graphene film, a comparative analysis was conducted on samples processed with and without this step (Figure S6a,b).

Figures 3g,h and S6c–f present SEM images at various magnifications of a Gr­(O)/Gr­(I)/SiO_2_/Pym Si substrate that was fabricated without the pre-etching step (Figure S6a). The low magnification images (Figures 3g and S6c–e) confirmed that the laminar flow-assisted etching method enabled the gentle and well-controlled removal of the catalytic Cu film for successfully producing conformal graphene films on the complex pyramidal microstructure. The graphene film exhibited good continuity and structural integrity with no observable detachment, folding, or displacement, but displayed several observable cracks (Figures 3g and S6e, black arrows). These cracks were primarily located in the valleys between adjacent pyramids and corresponded to the strip-like defects observed in the Cu film before etching (Figure 3c, black arrows). This spatial correlation suggests that the graphene cracks likely originated during CVD rather than metal etching, particularly in regions where the catalytic Cu layer was absent or discontinuous. High-magnification SEM images of the graphene film on the apex of a pyramid (Figures 3h and S6f) revealed that the graphene film retained a notable wrinkled morphology, consistent with the surface features observed on the Cu-covered sample before metal removal (Figure 3f). These wrinkles, caused by the mismatch in surface area between the overlying few-layer graphene and the underlying microstructures, prevented the film from forming complete intimate adherence with the substrate. Raman spectra collected from nine randomly selected positions across the Gr­(O)/Gr­(I)/SiO_2_/Pym Si substrate (Figure 3i) displayed the characteristic D (∼1350 cm^–1^), G (∼1581 cm^–1^), and 2D (∼2698 cm^–1^) bands of graphene. The average intensity ratio of the 2D and G bands (*I*_2D_/*I*_G_ ratio) was 0.81 ± 0.05, indicating that the produced graphene film had a thickness of 2–3 layers.^20,41^

By contrast, partial detachment of the Gr­(O) layer occurred when the pre-etching process was performed without conducting ultrasonication before rinsing the sample with DI water. Consequently, fragmented graphene flakes were observed on the underlying substrate after Cu removal was completed in the microfluidic etching process. These flakes appeared as randomly distributed dark patches across the pyramidal microstructures (Figure S7a). High-magnification SEM imaging revealed that beneath these dark patches (labeled as “outermost graphene film” in Figure S7b), a smooth, conformal graphene film remained on the underlying pyramidal substrate. This interfacial graphene film indicated in Figure S7b corresponded to the Gr­(I) layer grown at the Cu–SiO_2_ interface, confirming the presence of a multilayer graphene structure in samples lacking sufficient pre-etching. To ensure complete Gr­(O) removal and minimize contamination from deposited Gr­(O) fragments, a thorough pre-etching procedure was essential (Figure S6b). The adopted process involved immersing the Gr­(O)/Cu/Gr­(I)/SiO_2_/Pym Si substrates in 0.05 M APS for 5 min, following which they were subjected to ultrasonication in DI water for at least 10 min. This process resulted in the effective removal of the Gr­(O) layer and allowed the successful production of a uniform, monolayer graphene that conformally adhered to the SiO_2_/Pym Si substrate [Gr­(I)/SiO_2_/Pym Si in Figure 1] after the Cu film was completely removed by the laminar flow-assisted etching method.

Figure 4a presents a photograph of a representative sample obtained after CVD [a Gr­(O)/Cu/Gr­(I)/SiO_2_/Pym Si substrate], which displayed a metallic luster and no noticeable sign of Cu dewetting. After this Gr­(O)/Cu/Gr­(I)/SiO_2_/Pym Si substrate was subjected to thorough pre-etching and Cu removal, a Gr­(I)/SiO_2_/Pym Si substrate was obtained that exhibited a distinctly darker and uneven appearance (Figure 4b) compared with previously reported flat Gr/SiO_2_/Si substrates.^20^ The darker appearance suggested the presence of randomly distributed pyramidal microstructures, which can effectively suppress the reflection of incident light.^42^ The presence of pyramidal features was confirmed by optical microscopy (Figure 4f) and low-magnification SEM (Figure 4i). Raman spectra collected from nine positions on the Gr­(I)/SiO_2_/Pym Si sample (marked in Figure 4b) showed excellent spectral consistency (Figure 4c). These spectra contained characteristic peaks of graphene, including the D (∼1346 cm^–1^), G (∼1582 cm^–1^), and 2D (∼2689 cm^–1^) bands. Moreover, the relative peak intensities, such as the *I*_D_/*I*_G_ and *I*_2D_/*I*_G_ ratios, were consistent with values reported for monolayer graphene with low defect density.^20,36^ To assess the spatial uniformity and coverage of the graphene film, two-dimensional Raman mapping was performed over both macroscale and microscale regions. For the macroscale analysis, Raman measurements were conducted over a 0.7 × 0.7 cm^2^ area (within the 0.75 × 0.75 cm^2^ substrate shown in Figure 4b) using a spatial resolution of 14 × 14 pixels (0.5 mm/pixel). The resulting spatial distributions of the *I*_2D_/*I*_G_ (Figure S8a) and *I*_D_/*I*_G_ (Figure S8b) ratios provided strong evidence of uniform coverage across the scanned region. Statistical analysis of the 196 Raman spectra collected from this area revealed an average *I*_2D_/*I*_G_ ratio of 1.83 ± 0.38 (Figure S8c), with approximately 94% of the data points exceeding a value of 1.4 (Figure S8d), indicating that the graphene film is predominantly monolayer. Additionally, the average *I*_D_/*I*_G_ ratio was 0.28 ± 0.15 (Figure S8e), suggesting a low density of structural defects.

**Figure 4:**
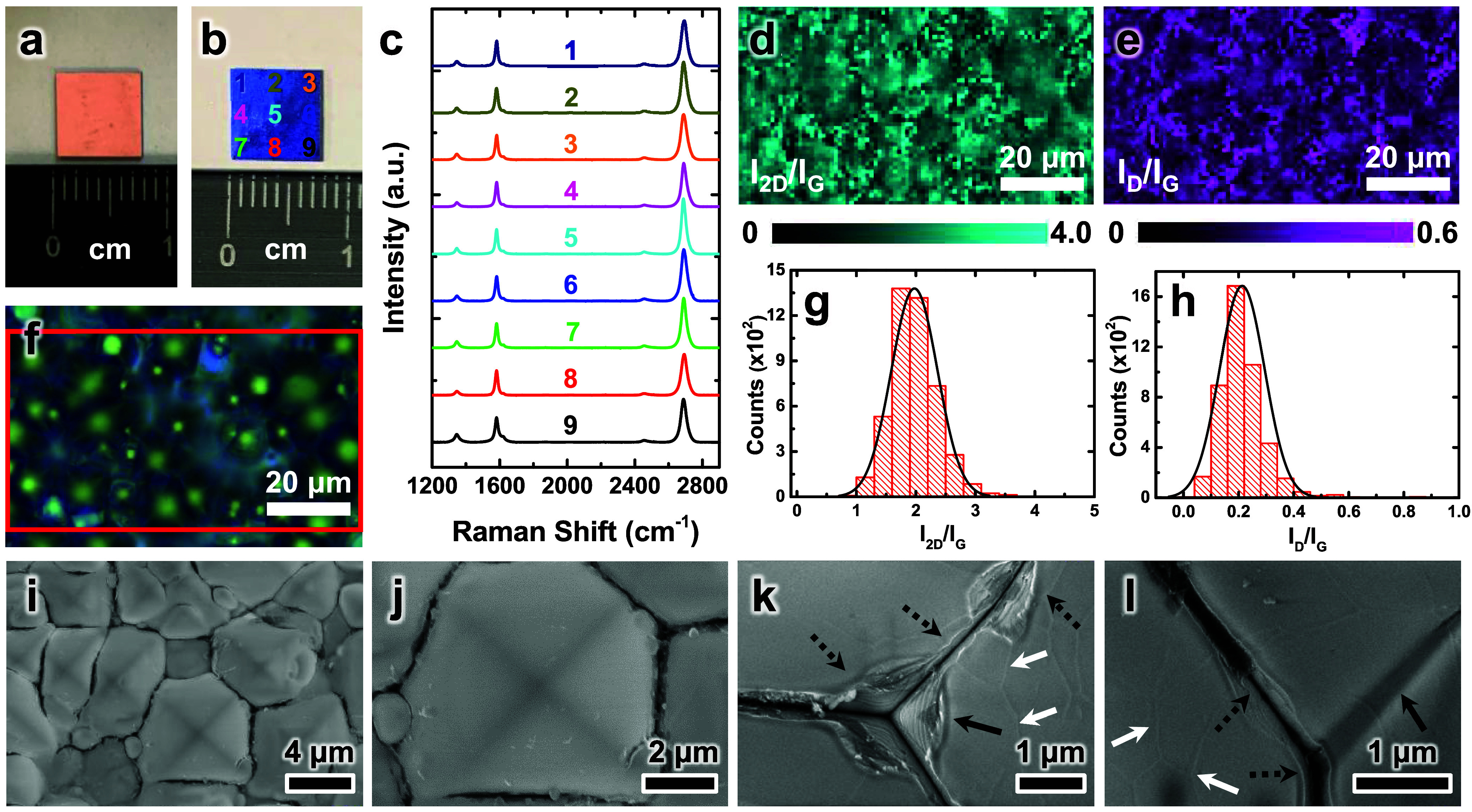
Photographs of a (a) Gr­(O)/Cu/Gr­(I)/SiO_2_/Pym Si substrate and (b) Gr­(I)/SiO_2_/Pym Si substrate. (c) Raman spectra acquired from nine locations on the Gr­(I)/SiO_2_/Pym Si substrate, as indicated in (b). (d, e) Two-dimensional Raman maps of the (d) *I*_2D_/*I*_G_ and (e) *I*_D_/*I*_G_ ratios for the region outlined by the red rectangle in (f). (f) An optical micrograph of the Gr­(I)/SiO_2_/Pym Si substrate. (g, h) Histograms of the statistical distributions of the (g) *I*_2D_/*I*_G_ and (h) *I*_D_/*I*_G_ ratios in (d) and (e), respectively. (i–l) SEM images of the Gr­(I)/SiO_2_/Pym Si substrate: (i) low-magnification overview, (j) single pyramid, and (k, l) basal joints between adjacent pyramids. In (k), graphene tears and folds are marked by dashed and solid black arrows, respectively. In (l), the solid black arrow indicates the conformal graphene film spanning continuously across the pyramidal valley, and the dashed black arrows indicate the conformal graphene film showing cracks along the valley. The white arrows in (k, l) indicate traces of Cu grain boundaries, which are commonly observed in transfer-free graphene grown at the Cu–SiO_2_ interface through CVD.

From microscale analysis, Raman mapping was performed over a 90 × 50 μm^2^ area (Figure 4f) with a spatial resolution of 90 × 50 pixels (1 μm/pixel). Absolute intensity maps of the 2D, G, and D bands acquired in a fixed focal plane are shown in Figure S9b–d. However, these unnormalized intensity maps inadequately represented the true graphene coverage because of the topographical variation of the pyramidal microstructures. Such variation resulted in focal mismatches and intensity fluctuations during Raman spectrum acquisition at a single focal depth.

To overcome this problem and accurately visualize the distribution of conformal graphene, Raman mapping of Si at 520.5 cm^–1^ was performed over the same area indicated by the red frame in Figure 4f. The mapping data of Si (Figure S9e) served as an internal standard for normalizing the Raman intensity of graphene bands. Corrected intensity maps of *I*_2D_/*I*_Si_, *I*_G_/*I*_Si_, and *I*_D_/*I*_Si_ were obtained by calibrating the 2D, G, and D intensities to the Si signal, confirming the conformal coverage of the graphene layer over the entire pyramidal surface (Figure S9f–h). Spatial maps of the *I*_2D_/*I*_G_ (Figure 4d) and *I*_D_/*I*_G_ (Figure 4e) ratios provided further evidence of high structural homogeneity across the substrate. Statistical analysis of 4500 Raman spectra obtained from two-dimensional maps revealed an average *I*_2D_/*I*_G_ ratio of 1.98 ± 0.65 (Figure 4g), with approximately 95% of the data points exceeding 1.4 (Figure S10a), indicating that the graphene film primarily had monolayer thickness. The average *I*_D_/*I*_G_ ratio was 0.21 ± 0.08 (Figure 4h), and the full width at half-maximum values of the 2D and G bands were 37.8 ± 5.6 cm^–1^ (Figure S10b,c) and 20.5 ± 6.9 cm^–1^ (Figure S10d), respectively. These results further confirmed the uniform monolayer nature and low defect density of the produced graphene at higher spatial resolution. Taken together, the results from both macroscale and microscale Raman analyses demonstrated that the synthesized Gr­(I)/SiO_2_/Pym Si substrate was uniformly covered by a high-quality graphene monolayer with a low density of structural defects.

Figure 4i–l display SEM images of a representative Gr­(I)/SiO_2_/Pym Si substrate at different magnifications, highlighting its distinct morphological features compared with those of substrates containing continuous Gr­(O) films (Figure 3g,h) or fragmented Gr­(O) flakes (Figure S7a,b). The Gr­(I)/SiO_2_/Pym Si substrate retained well-defined pyramidal microstructures (Figure 4i), showing smooth and clean facets free of visible contaminations, such as nanoparticles or wrinkled graphene debris (Figure 4j). At some basal joints between pyramids, features resembling tears (Figure 4k, dashed black arrows) and folds (Figure 4k, solid black arrow) were observed, implying the existence of a graphene film, which was confirmed by Raman characterizations (Figure 4c–e). This film-like structure either extended continuously across the valleys between adjacent pyramids (Figure 4l, solid black arrow) or exhibited cracks along these valleys (Figure 4l, dashed black arrows), suggesting that the continuity of the graphene layer was affected by the integrity of the Cu film during CVD. In regions with notable Cu sublimation and dewetting, particularly in the pyramidal valleys (Figure 3c, black arrows), the catalytic growth of graphene was inhibited, resulting in discontinuities in the graphene film. Moreover, residual traces of Cu grain boundaries were observed in the conformal graphene film (Figure 4k,l, white arrows). These residual features are commonly observed in transfer-free graphene grown at a Cu–SiO_2_ interface through CVD.^20,43,44^

To verify the presence of the Gr­(I) layer, X-ray photoelectron spectroscopy (XPS) analysis was performed for Gr­(I)/SiO_2_/Pym Si and bare SiO_2_/Pym Si substrates. The XPS survey spectra of both samples contained detectable carbon signals; however, the atomic percentage of carbon was substantially higher in the Gr­(I)/SiO_2_/Pym Si substrate (Figure S11a,b), suggesting the presence of additional carbon materials (i.e., graphene) in this substrate. By contrast, the weak carbon signal from the bare SiO_2_/Pym Si substrate was attributed to adventitious carbon, which refers to the ubiquitous carbonaceous contamination found on most air-exposed surfaces. Deconvolution of the high-resolution C 1s spectra supported these assumptions. The C 1s spectrum of the SiO_2_/Pym Si substrate was primarily composed of a peak related to alkyl carbon (C–C/C–H) at 284.8 eV and peaks related to oxygen-containing species, such as hydroxyl (C–OH), epoxide/ether (C–O–C), carbonyl (C=O), and carboxyl (O=C–OH) groups (Figure S11c). These results correspond to the presence of adsorbed ambient contaminants. By contrast, the C 1s spectrum of the Gr­(I)/SiO_2_/Pym Si substrate was dominated by a peak related to graphite-like sp^2^ carbon (C=C/C–C) at 284.5 eV, exhibiting an asymmetric line shape characteristic of graphene (Figure S11d). This result confirmed the successful formation of a graphene film on the pyramidal substrate.

Based on the above characterizations, our method produced a highly uniform and conformal coating composed of ∼95% monolayer and ∼5% bilayer graphene, with low defect density on micropyramidal 3D structures. Compared with representative pioneering studies, our approach offers superior graphene quality, precise layer control on complex 3D microstructures, and mild processing conditions, demonstrating a novel and competitive advance in the field of direct graphene synthesis (Table S1).

### 3.3 Evaluation of the SERS Performance of Conformal Graphene-Coated Pyramidal Substrates

The conformal graphene-coated pyramidal substrates [Gr­(I)/SiO_2_/Pym Si] were employed for SERS detection using R6G as a model probe molecule. To elucidate the contributions of various surface modifications to the SERS activity, we examined the Raman responses of four types of substrates: flat silicon (SiO_2_/Flat Si), pyramidal silicon (SiO_2_/Pym Si), graphene-coated flat silicon [Gr­(I)/SiO_2_/Flat Si], and graphene-coated pyramidal silicon [Gr­(I)/SiO_2_/Pym Si]. The Raman intensity scale was expressed in arbitrary units (a.u.), with numerical values retained to facilitate evaluation of intensity variations and statistical analysis. For a clear comparison of SERS performance, stacked Raman spectra collected from these substrates were presented in Figure 5a. To facilitate the identification of weak R6G peaks that may be obscured in the stacked view, individually separated Raman spectra for each substrate are provided in Figure S12 in the Supporting Information. At an R6G concentration of 10^–5^ M, negligible Raman signals were acquired from the substrates lacking graphene coverage, regardless of the presence of pyramidal microstructures (Figure 5a, green and blue lines and Figure S12a,b). Previous studies have reported that well-distributed pyramidal microstructures can induce local enhancement of incident light by promoting multiple internal reflections and effective laser oscillation within the pyramidal valleys.^45,46^ However, such localized enhancement alone was insufficient to produce detectable Raman signals of R6G at a concentration of 10^–5^ M. The Raman spectra of R6G on the SiO_2_/Flat Si and SiO_2_/Pym Si substrates only contained a characteristic Si peak at 520.5 cm^–1^.

**Figure 5:**
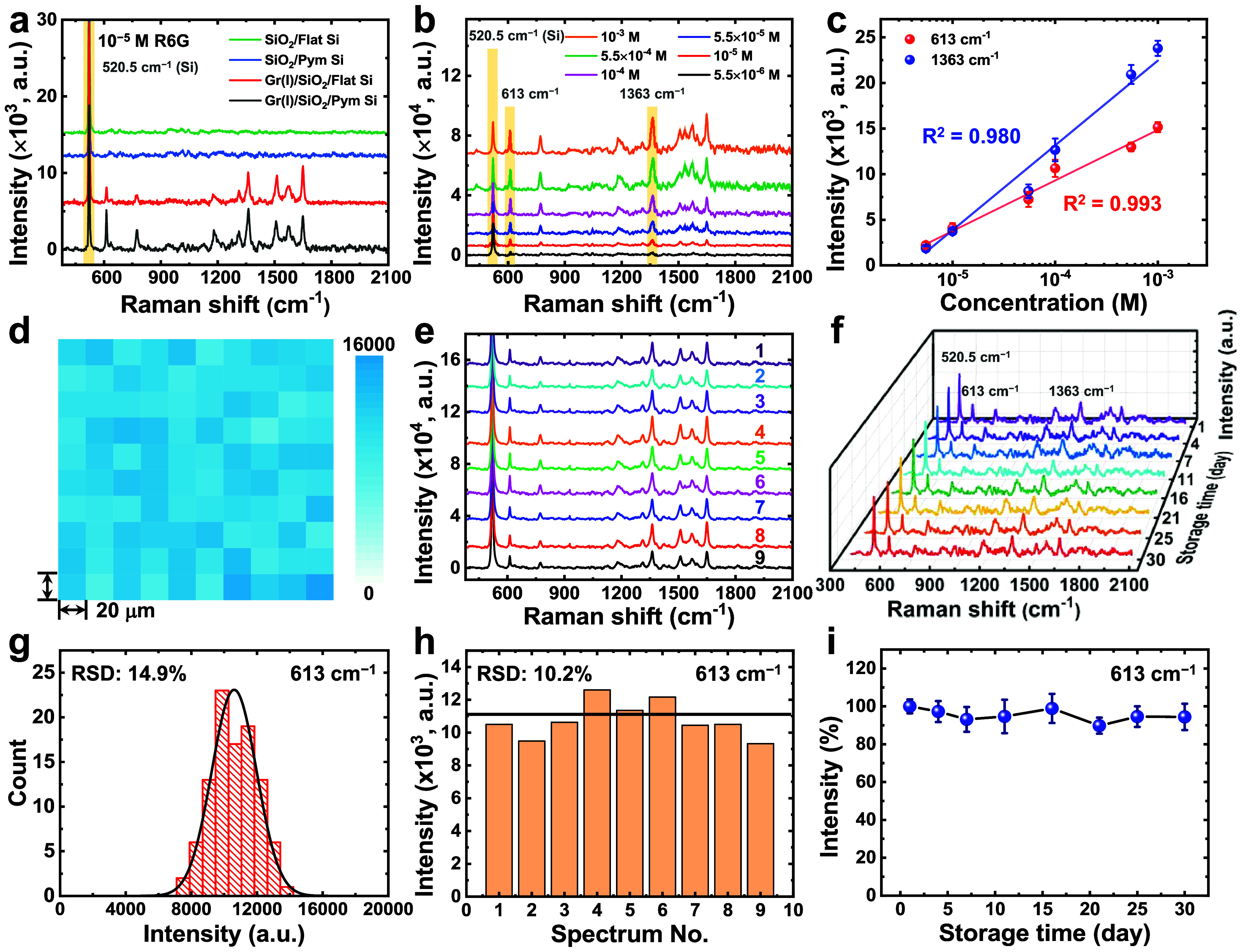
(a) Raman spectra of 10^–5^ M R6G on SiO_2_/Flat Si, SiO_2_/Pym Si, Gr­(I)/SiO_2_/Flat Si, and Gr­(I)/SiO_2_/Pym Si substates. (b) SERS spectra for various concentrations of R6G on the Gr­(I)/SiO_2_/Pym Si substates. (c) The SERS signal intensities at 613 and 1363 cm^–1^ were plotted as a function of the R6G concentration on a logarithmic scale. (d–f) Data recorded for 10^–4^ M R6G on a Gr­(I)/SiO_2_/Pym Si substrate: (d) a two-dimensional intensity map for the peak at 613 cm^–1^ over a 200 × 200 μm^2^ area with a 20 μm step size; (e) SERS spectra over a 6.0 × 6.0 mm^2^ area with a 2 mm step size; and (f) SERS spectra measured over 30 days on the substrate stored under 40% relative humidity. (g) Histogram of the intensity distribution of measurements shown in (d). (h) Bar chart of the intensity at 613 cm^–1^ in Raman spectra shown in (e), with the average value marked by a black line. (i) Changes in the intensity at the 613 cm^–1^ peak as a function of time determined from (f), indicating the long-term stability of substrate performance.

By contrast, the graphene-coated silicon substrates exhibited notable SERS enhancement under identical conditions, displaying distinct Raman peaks at 613 (in-plane bending of the C–C ring), 773 (out-of-plane bending of the C–H bond), 1182 (in-plane bending of the C–H bond), 1311 (hybrid vibration associated with aromatic rings and the NHC_2_H_5_ group), 1363, 1510, 1573, and 1649 cm^–1^ (Figure 5a, red and black lines and Figure S12c,d). The peaks at 1363, 1510, 1573, and 1649 cm^–1^ are attributed to aromatic C–C stretching modes.^47,48^ The enhanced Raman signals observed for the Gr­(I)/SiO_2_/Flat Si and Gr­(I)/SiO_2_/Pym Si substrates can be primarily attributed to the graphene-enhanced Raman scattering (GERS) effect.^29^ Previous studies have shown that fluorescent dyes, such as R6G, undergo significant fluorescence quenching upon adsorption onto graphene due to rapid resonance energy transfer from the dye molecules to the graphene surface. This effect substantially reduces background fluorescence and enables the detection of clear Raman signals of R6G, even under challenging conditions such as near-resonance excitation using a 532 nm laser.^27,49^ Furthermore, π–π interactions between graphene and R6G not only promote molecular enrichment but also restrict molecular vibrational motion. This confinement reduces vibrational amplitudes and multimode coupling among adjacent R6G molecules, thereby suppressing self-absorption and enhancing Raman signal intensity.^50,51^ Notably, charge-transfer interactions are also believed to play a critical role in the GERS effect. Upon contact with graphene, R6G molecules undergo electron redistribution to establish a new interfacial equilibrium, which increases their polarizability and thus enhances the Raman scattering intensity.^31,52^ Moreover, Raman excitation profile analyses and band alignment studies have shown that the enhanced Raman signals in the GERS system rely on ground-state charge-transfer mechanisms.^51,53^

However, the efficiency of this charge transfer is governed by several interrelated factors, including energy-level alignment between the molecule and graphene,^31,53–55^ molecular orientation,^56^ graphene doping level,^31,53–55^ molecule–graphene distance,^56^ and laser excitation wavelength.^51^ These complex variables make mechanistic analysis particularly challenging. Nevertheless, our substrates provide a graphene-only SERS platform that holds promise for deconvoluting these interrelated variables and advancing the fundamental understanding of GERS effects. Such a mechanistic exploration, however, lies beyond the scope of this application-oriented study and would be more appropriately addressed in a separate, fundamentally focused investigation.

To quantitatively evaluate the SERS performance of our substrates, we employed the analytical enhancement factor (AEF),^57,58^ a practical metric that complements the traditional enhancement factor by assessing signal amplification under realistic experimental conditions. The AEF is expressed as follows^57,58^

5 $$AEF=\frac{I_{SERS}/C_{SERS}}{I_{Raman}/C_{Raman}}$$

where *I*_SERS_ refers to the SERS signal intensity for an analyte at a concentration of *C*_SERS_ on a SERS-active substrate and *I*_Raman_ represents the general Raman intensity for the same analyte in non-SERS conditions at a potentially different concentration *C*_Raman_. Raman spectra were recorded under identical measurement conditions for 10^–3^ M R6G on the SiO_2_/Flat Si substrates and for 10^–5^ M R6G on the Gr­(I)/SiO_2_/Flat Si and Gr­(I)/SiO_2_/Pym Si substrates (Figure S13). The Gr­(I)/SiO_2_/Flat Si substrates exhibited AEFs ranging from 5.7 to 18.6 for different Raman peaks (Table S2). These values were consistent with the chemical mechanism (CM)-based SERS process, which exhibits vibration-dependent behavior and generally results in moderate enhancement, with AEFs below the typical upper limit of ∼100. Among these substrates, the Gr­(I)/SiO_2_/Pym Si substrates exhibited the highest AEF values, ranging from 19.5 to 45.5 (Figure 5a and Table S2), which highlighted the synergistic SERS enhancement arising from the pyramidal microstructures and the graphene-mediated CM-based mechanism.

To assess the detection capability of the Gr­(I)/SiO_2_/Pym Si substrate, SERS spectra were collected across a range of R6G concentrations (Figure 5b). To ensure that all spectra were acquired under consistent and well-focused conditions, a software-based autofocus system was employed to reoptimize focus at each measurement point; details are provided in Section 4 of the Supporting Information. The concentration range was selected to demonstrate the linear dynamic response of the substrate under consistent detection conditions. Measurements were conducted using a 532 nm laser (0.25 mW, 0.5% of full scale) focused through a 100× objective, with an exposure time of 1.0 s per point. Due to signal saturation at higher concentrations, the upper concentration limit was set at 10^–3^ M. At the lower end, characteristic Raman signals of R6G were no longer detectable below 10^–6^ M, as shown in Figure S14a; thus, these concentrations were excluded from Figure 5b,c. Among the observed peaks, the bands at 613 and 1363 cm^–1^ were selected for calibration because of their strong intensity and reproducibility. As displayed in Figure 5c, the SERS intensities at these peaks exhibited excellent linear correlations with the R6G concentration on a logarithmic scale, with correlation coefficients (*R* ^2^) of 0.993 and 0.980 at 613 and 1363 cm^–1^, respectively. Compared with the signal at 1363 cm^–1^, the 613 cm^–1^ peak showed a better linear correlation with the R6G concentration and a higher AEF value (Table S2). Therefore, the signal at 613 cm^–1^ was used as the primary reference for evaluating key sensing parameters, including signal reproducibility and substrate stability. The limits of detection (LOD),^59^ calculated as 3.3 times the standard deviation of the blank divided by the slopes of the calibration curves in Figure 5c, were 2.5 × 10^–6^ M at 613 cm^–1^ and 4.3 × 10^–6^ M at 1363 cm^–1^.

The signal reproducibility at various positions on a SERS substrate indicates the uniformity of SERS activity across the surface. To examine this sensing parameter, we conducted spatially resolved Raman mapping by using 10^–4^ M R6G on the Gr­(I)/SiO_2_/Pym Si substrate across two scales: a microscale area of 200 × 200 μm^2^ with a 20 μm step size and a macroscale area of 6.0 × 6.0 mm^2^ with a 2 mm step size. The two-dimensional Raman intensity map for the 613 cm^–1^ peak revealed high uniformity in signal intensity across the microscale region (200 × 200 μm^2^, Figure 5d), with the relative standard deviation (RSD) across 100 measurement points being 14.9% (Figure 5g). The detailed method for calculating the RSD values, including the corresponding equations (eqs S1–S3), is provided in Section 5 of the Supporting Information. In the macroscale region (6.0 × 6.0 mm^2^), Raman spectra were recorded at nine positions across the substrate surface. These spectra exhibited high consistency (Figure 5e), with the RSD of 10.2% (Figure 5h). Both RSD values were below the widely accepted threshold of 20% for SERS substrates with high sensing reproducibility and uniform activity in practical applications.^60^ These findings confirmed the high consistency of the SERS signals recorded on the Gr­(I)/SiO_2_/Pym Si substrate, making it well-suited for quantitative SERS analysis. Substrate stability is another critical factor in SERS applications. We assessed this factor for the Gr­(I)/SiO_2_/Pym Si substrate by recording SERS spectra for 30 days. During this time, the substrate was stored under controlled conditions, with the relative humidity being 40%, and Raman measurements were performed in an ambient environment. As depicted in Figure 5f, the Raman spectra recorded for 10^–4^ M R6G exhibited minimal variations, with the peak intensity at 613 cm^–1^ remaining highly stable throughout the testing period (Figure 5i). This excellent long-term stability of the SERS response was attributed to the chemical stability and oxidation resistance of the graphene coating, as well as the structural robustness of the silicon-based micropyramidal features.

### 3.4 Optimization of AgNP Deposition on Graphene-Coated Pyramidal Silicon Substrates for Enhancing SERS Activity

In conventional SERS measurements, the enhancement factor contributed by CM is typically less than 100. By contrast, the electromagnetic mechanism (EM) can boost Raman signals by a factor ranging from 10^4^ to 10^9^.^61^ The signal enhancement caused by the EM is attributed to the locally enhanced electromagnetic fields generated by surface plasmon resonance, which occurs when incident light interacts with plasmonic materials, such as noble metal nanoparticles. To improve the SERS performance of Gr­(I)/SiO_2_/Pym Si substrates, which was dominated by the graphene-mediated CM-based mechanism, AgNPs were deposited on the substrates’ surface to introduce EM-based enhancement, thereby improving the overall sensing capability through the synergistic effects of EM and CM enhancements. AgNPs of different sizes were deposited on the Gr­(I)/SiO_2_/Pym Si substrates by adjusting the deposition time during e-beam evaporation, producing AgNPs/Gr­(I)/SiO_2_/Pym Si substrates. The presence of silver on this type of substrate was confirmed by its XPS survey spectrum (Figure S15a), and the high-resolution Ag 3d XPS spectrum revealed the metallic nature and high purity of the deposited AgNPs, which showed no detectable oxidation (Figure S15b). The morphologies of the AgNPs produced under different deposition durations were characterized through SEM imaging, and the particle dimensions were determined using ImageJ (Figure 6a–e).^39^ Histograms of the particle size distributions indicated that the average diameter of the AgNPs increased progressively from 10.5 to 50.5 nm as the deposition time was extended from 50 to 300 s (Figures 6f and S16). Moreover, notable particle aggregation was observed when the deposition time exceeded 250 s.

**Figure 6:**
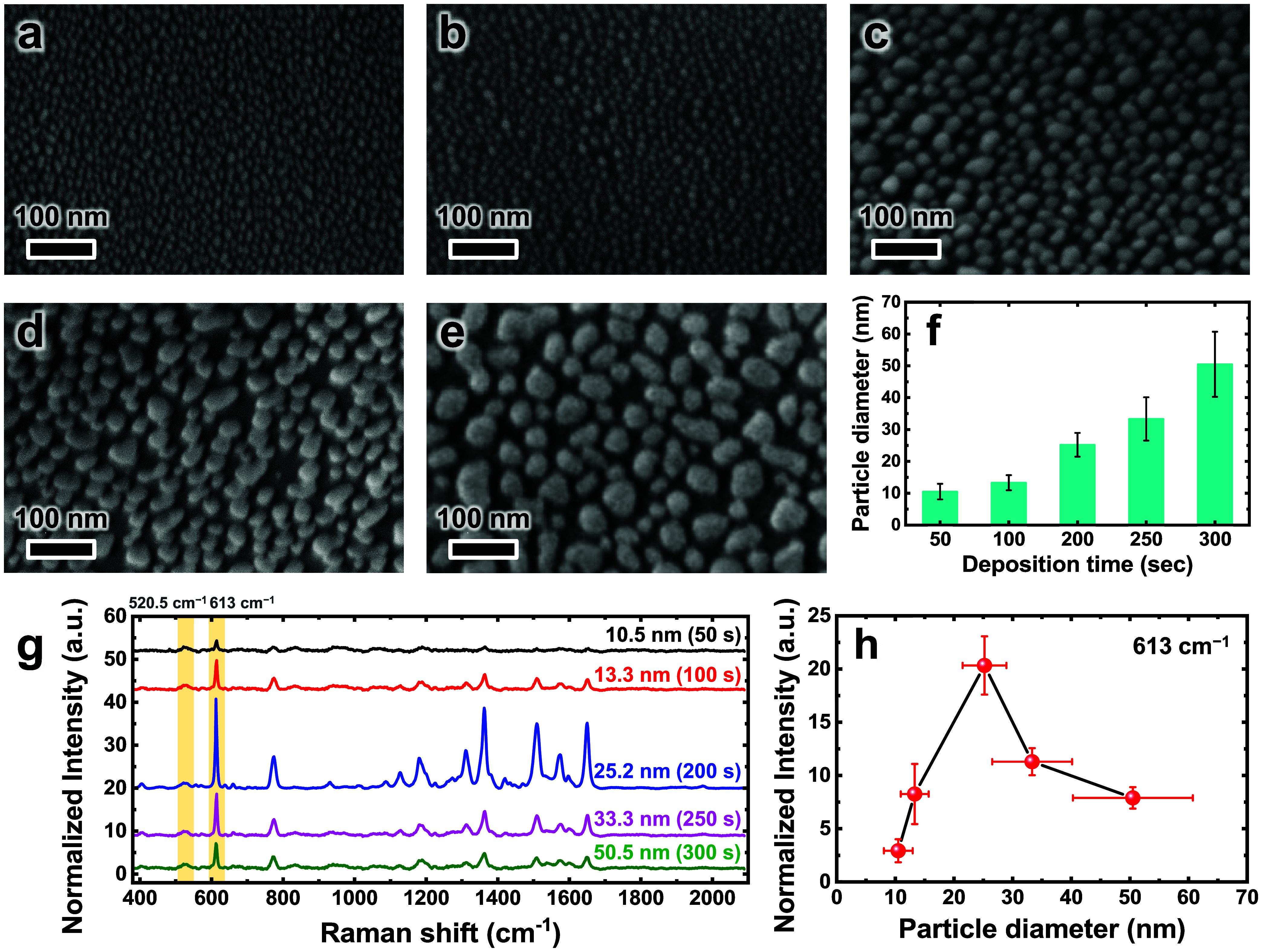
SEM images of AgNPs deposited through e-beam evaporation for (a) 50, (b) 100, (c) 200, (d) 250, and (e) 300 s. (f) Average diameters of AgNPs as a function of deposition time, derived from (a–e). (g) SERS spectra of 10^–7^ M R6G collected from AgNPs/Gr­(I)/SiO_2_/Pym Si substrates, where AgNPs of varying diameters were produced by different deposition times. (h) Plot of SERS intensity at 613 cm^–1^ as a function of AgNP diameter. The highest SERS response was observed for AgNPs with an average diameter of 25.2 ± 3.7 nm, corresponding to a deposition time of 200 s.

To identify the optimal deposition time and consequently the ideal size of AgNPs for maximizing SERS performance, we evaluated the SERS spectra of 10^–7^ M R6G on AgNPs/Gr­(I)/SiO_2_/Pym Si substrates, where AgNPs of varying diameters were prepared using different deposition times (Figure 6g). The Raman intensity at 613 cm^–1^ was plotted as a function of AgNP diameter (Figure 6h). This analysis revealed that the strongest SERS response occurred on the AgNPs/Gr­(I)/SiO_2_/Pym Si substrate containing AgNPs with an average diameter of 25.2 ± 3.7 nm, corresponding to a deposition time of 200 s (Figure 6f–h). According to numerical simulation studies, under a constant interparticle distance, larger plasmonic nanoparticles can generate stronger localized electric fields at their edges, thereby enhancing SERS signals through the EM process.^62^ However, additional factors, such as the density of high-field regions (i.e., hot spot density) and the gap sizes between adjacent particles, also play critical roles in determining the overall contribution of the EM to SERS enhancement.^63–66^ Therefore, the enhanced SERS performance exhibited by the substrate with AgNPs having a size of 25.2 nm likely reflects a favorable balance among particle size, interparticle distance, and the density of high-field regions. Based on these findings, AgNPs/Gr­(I)/SiO_2_/Pym Si substrates fabricated with 25.2 nm AgNPs (deposited for 200 s via e-beam evaporation) were selected for subsequent SERS experiments. These optimized substrates combine the CM enhancement of graphene with the strong EM of well-dispersed AgNPs, thus serving as highly efficient platforms for sensitive SERS detection.

### 3.5 Assessment of the SERS Performance of AgNPs/Graphene-Coated Pyramidal Silicon Substrates

Figure 7a illustrates the SERS spectra of 10^–7^ M R6G on SiO_2_-coated, pyramid-textured silica substrates subjected to three different surface modifications: conformal graphene coating [Gr­(I)/SiO_2_/Pym Si], AgNP deposition (AgNPs/SiO_2_/Pym Si), and AgNP deposition on a conformal graphene coating [AgNPs/Gr­(I)/SiO_2_/Pym Si]. These spectra indicated that the CM enhancement originating from graphene alone was insufficient for detecting 10^–7^ M R6G. By contrast, the EM enhancement arising from AgNPs substantially enhanced the SERS response, with an AEF value of 6.81 × 10^3^ for the 613 nm^–1^ Raman peak (Table S2). Moreover, the synergistic effect of CM and EM in the AgNPs/Gr­(I)/SiO_2_/Pym Si substrate further enhanced the AEF value to 1.08 × 10^5^ for detecting 10^–8^ M R6G (Table S2). In addition to enhanced signal intensity, the conformal graphene coating of the AgNPs/Gr­(I)/SiO_2_/Pym Si substrate effectively suppressed the intrinsic fluorescence of silver, as indicated by the raw spectra without background subtraction shown in Figure S17. This notable fluorescence quenching effect of graphene further improved the overall sensing performance.^27,46^

**Figure 7:**
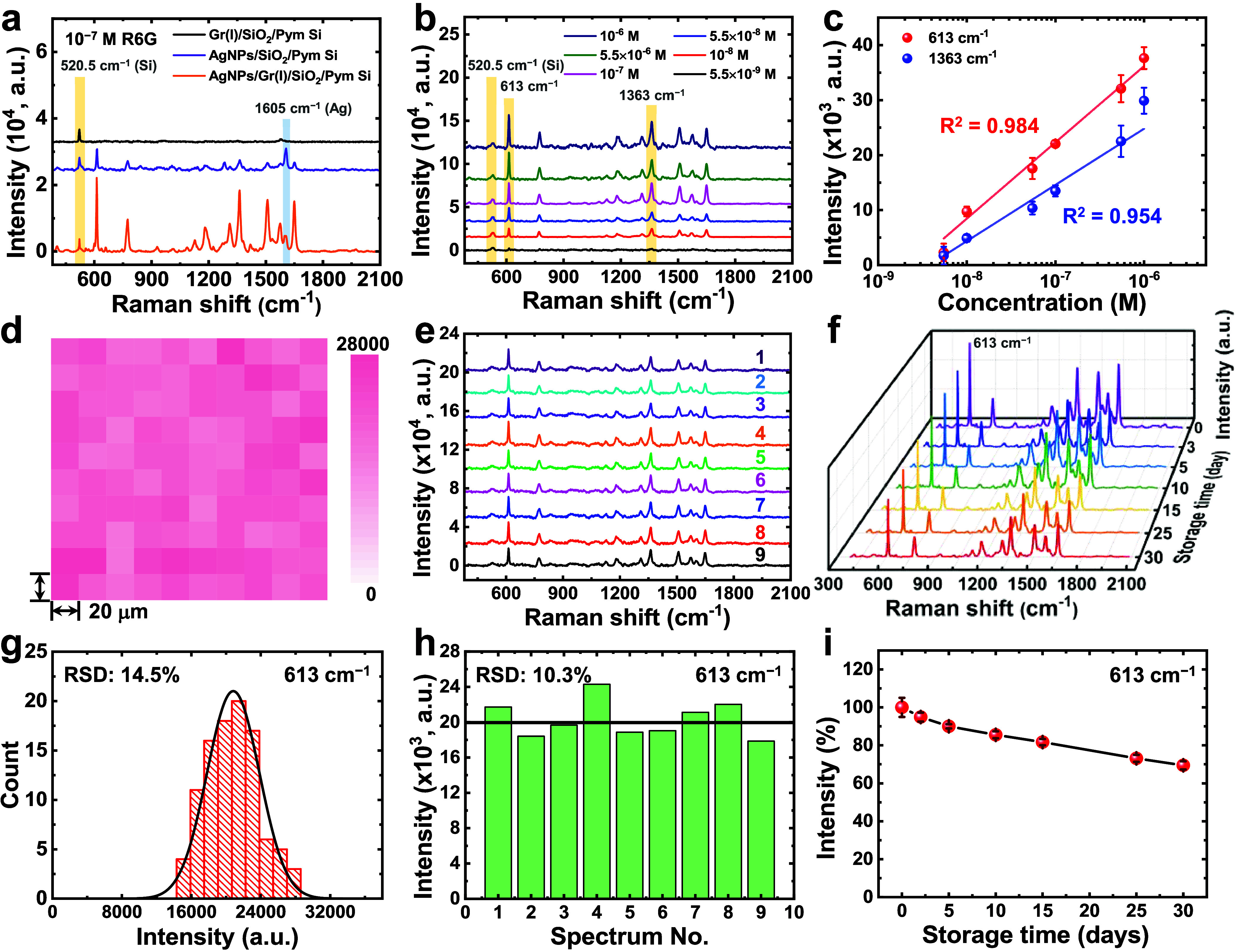
(a) SERS spectra of 10^–7^ M R6G on Gr­(I)/SiO_2_/Pym Si, AgNPs/SiO_2_/Pym Si, and AgNPs/Gr­(I)/SiO_2_/Pym Si substates. (b) SERS spectra for various concentrations of R6G on the AgNPs/Gr­(I)/SiO_2_/Pym Si substates. (c) The SERS signal intensities at 613 and 1363 cm^–1^ were plotted as a function of the R6G concentration on a logarithmic scale. (d–f) Data recorded for 10^–7^ M R6G on a AgNPs/Gr­(I)/SiO_2_/Pym Si substrate: (d) two-dimensional intensity map for the peak at 613 cm^–1^ over a 200 × 200 μm^2^ region with a 20 μm step size; (e) SERS spectra obtained across a 6.0 × 6.0 mm^2^ area with a 2 mm step size; and (f) SERS spectra measured over 30 days on the substrate stored in a vacuum desiccator. (g) Histogram of the intensity distribution of measurements shown in (d). (h) Bar chart of the intensities at 613 cm^–1^ in Raman spectra shown in (e), with the average value marked by a black line. (i) Changes in the intensity at the 613 cm^–1^ peak as a function of time determined from (f), indicating the long-term stability of substrate performance.

To assess the detection sensitivity of the AgNPs/Gr­(I)/SiO_2_/Pym Si substrate, the SERS spectra of R6G at various concentrations were recorded (Figure 7b). To avoid signal saturation, the upper concentration limit was set at 10^–6^ M. At the low concentration of 5.5 × 10^–9^ M, although the signal is weak and close to the noise level, the characteristic Raman peaks at 613 and 1363 cm^–1^ were still discernible (Figure S14b). As shown in Figure 7c, the intensities of these peaks exhibited clear linear relationships with the logarithm of R6G concentration, with *R* ^2^ values of 0.984 and 0.954 at 613 and 1363 cm^–1^, respectively. The LOD values, calculated as 3.3 times the standard deviation of the blank divided by the slopes of the calibration curves in Figure 7c, were 3.2 × 10^–9^ M at 613 cm^–1^ and 5.4 × 10^–9^ M at 1363 cm^–1^. These results confirmed that the AgNPs/Gr­(I)/SiO_2_/Pym Si substrate enabled reliable detection of R6G at concentrations as low as 5.5 × 10^–9^ M, which was 3 orders of magnitude lower than the detection limit of the Gr­(I)/SiO_2_/Pym Si substrate. To evaluate the spatial uniformity and temporal stability of the AgNPs/Gr­(I)/SiO_2_/Pym Si substrate, the Raman intensity at the 613 cm^–1^ peak was analyzed. This intensity was selected for its better linear relationship with the R6G concentration and higher AEF value for detecting R6G. Raman mapping was performed at two scales by using 10^–7^ M R6G: a microscale 200 × 200 μm^2^ area with a 20 μm step size and a macroscale 6.0 × 6.0 mm^2^ area with a 2 mm step size. The resulting maps showed high uniformity, with the RSD values for the microscale and macroscale areas being 14.5% (Figure 7d,g) and 10.3% (Figure 7e,h), respectively. These RSD values are within the threshold of <20% for substrates with highly uniform SERS activity. These results underscore the suitability of the AgNPs/Gr­(I)/SiO_2_/Pym Si substrate for reliable quantitative SERS analysis. The temporal stability of this substrate was examined over 30 days under two storage conditions: 40% relative humidity and vacuum desiccation. The SERS spectra recorded in ambient environment for 10^–7^ M R6G indicated that although the Raman peak positions remained consistent on the substrates stored in both conditions (Figures 7f and S18a), the signal intensity declined notably for the substrate stored under 40% relative humidity, with the signal of R6G nearly disappearing by day 30 (Figure S18b). By contrast, for the substrate stored under vacuum desiccation, the signal intensity exhibited a gradual reduction of approximately 30% over 30 days (Figure 7i). This degradation in the detection performance was attributed to the susceptibility of AgNPs to oxidation during storage and measurement. Therefore, although vacuum desiccation extended operational lifespan, the AgNPs/Gr­(I)/SiO_2_/Pym Si substrate exhibited only moderate durability under practical conditions.

Moreover, we investigated their reusability using 10^–7^ M R6G. As shown in Figure S19a, the initial Raman spectrum recorded from a freshly prepared substrate exhibited distinct R6G signals with a low noise level, and the corresponding SEM image confirmed a uniform AgNPs/Gr­(I) coating on the micropyramidal Si surface (Figure S19b). After mild cleaning with deionized water, weak R6G signals remained, likely due to strong π–π interactions between R6G and the graphene surface (Figure S19c). While subsequent rigorous cleaning using acetone and 3 min sonication effectively removed most R6G signals (Figure S19d), the reused substrate exhibited diminished signal intensity and increased background noise upon redeposition of R6G (Figure S19e). SEM analysis revealed partial detachment of the AgNPs/Gr­(I) layer from the microstructures, indicating mechanical damage during the cleaning process (Figure S19f). These findings suggest that, in its current form, the substrate lacks sufficient reusability, and future optimization will be necessary to improve its mechanical robustness for repeated use.

To further demonstrate the practical application of the optimized AgNPs/Gr­(I)/SiO_2_/Pym Si substrates, we conducted preliminary detection experiments using four representative fungicides: thiram, fludioxonil, thiabendazole, and malachite green. All four analytes exhibited clear and concentration-dependent SERS signals, with detection limits reaching as low as 10^–8^ M and strong linearity (*R* ^2^ ≥ 0.985) across the tested concentration ranges. These results indicated the broad sensing capability and high sensitivity of the developed SERS platform beyond the model analyte R6G. The preliminary SERS spectra and calibration data for these fungicides are provided in the Supporting Information (Figure S20), while a comprehensive investigation of their detection and spectral characteristics is being prepared for a separate publication.

### 3.6 SERS Mechanism of the AgNPs/Gr­(I)/SiO_2_/Pym Si Substrate

Mechanisms that contribute to the SERS performance of the AgNPs/Gr­(I)/SiO_2_/Pym Si substrate are illustrated in Figure 8. The engineered pyramidal microstructures increase light trapping and specific surface area (Figure 8a). The conformal graphene coating promotes R6G adsorption via strong π–π interactions and effectively quenches the fluorescence of both R6G and AgNPs. This fluorescence suppression occurs through a Förster resonance energy transfer (FRET)-like mechanism, wherein the excited R6G molecule transfers its energy to graphene, followed by nonradiative relaxation of the excited electron to the Fermi level (*E*_F_) of graphene (Figure 8b).^27,49^ Consequently, the fluorescence signal of R6G is significantly reduced even under near-resonance excitation using a 532 nm laser (2.33 eV). Close molecular proximity to the graphene surface, achieved by enhanced molecular adsorption on graphene, further facilitates this FRET-like process.

**Figure 8:**
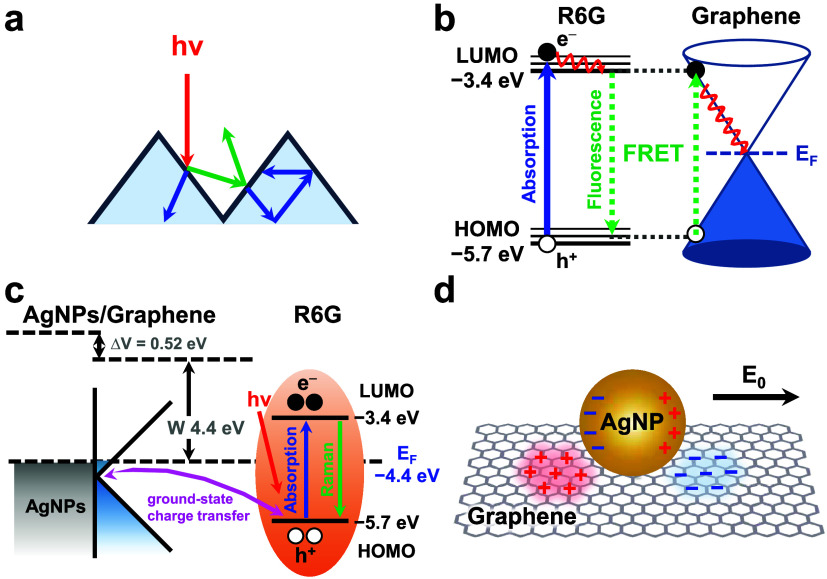
(a) Enhanced light trapping on pyramid-textured surface due to multiple reflections (green arrows) and light transmission (blue arrows) of an incident light (red arrow). (b) Graphene-mediated fluorescence quenching of R6G via a FRET-like mechanism. (c) Ground-state charge transfer between the AgNPs/graphene system (*E*_F_) and R6G (HOMO) increases molecular polarizability, leading to enhanced Raman scattering. (d) LSPs of AgNPs generate oscillating dipoles and local electric fields on graphene. The two-dimensional confinement of LSP on graphene leads to a reduced LSP wavelength and an enhanced reflected electromagnetic field, further amplifying the SERS signal. *E*_0_ refers to the electric field of incident light.

Raman signal enhancement on graphene-based substrates is often attributed to chemical enhancement via charge-transfer mechanisms. In our case, photoinduced (excited-state) charge transfer from the Fermi level (*E*_F_) of graphene (∼−4.6 eV) to the lowest unoccupied molecular orbital (LUMO, −3.4 eV) of R6G is unlikely under 532 nm excitation, due to a significant energy mismatch (1.2 eV gap vs 2.33 eV photon energy).^31^ Moreover, Raman excitation spectroscopy studies of graphene-enhanced Raman scattering have shown that changes in excitation wavelength can lead to enhancement variations that are consistent with ground-state charge-transfer mechanisms.^52^ Therefore, ground-state charge transfer between the highest occupied molecular orbital (HOMO, −5.7 eV) of R6G and graphene (*E*_F_, −4.6 eV) is more likely and dominates the enhancement mechanism (Figure S21a).^31,51^ Upon deposition of AgNPs, graphene experiences n-type doping, shifting its E_F_ from approximately −4.6 to −4.4 eV.^53,67,68^ The origin of this *E*_F_ shift in the AgNPs/graphene hybrid system is discussed in detail in Section 6 of the Supporting Information (Figure S21b). Since charge transfer is sensitive to band alignment, a closer alignment between R6G (HOMO) and the *E*_F_ of SERS substrates facilitates a stronger orbital hybridization and thus more efficient charge transfer. Based on this principle, the AgNPs/graphene hybrid (*E*_F_, −4.44 eV) may offer slightly poorer alignment with R6G (HOMO, −5.7 eV) compared to pristine graphene (*E*_F_, −4.60 eV), potentially reducing charge transfer efficiency. However, a recent work indicates that for R6G detection, minor shifts in the E_F_ of graphene-based substrates have only a limited effect on Raman intensity.^53^ Importantly, the *E*_F_ of the AgNPs/graphene hybrid remains near the center of R6G’s frontier orbital gap (HOMO at −5.7 eV and LUMO at −3.4 eV), which still favors significant ground-state charge transfer and results in a strong graphene-enhanced Raman scattering (GERS) effect (Figures 8c and S21c).^30^

Furthermore, AgNPs support localized surface plasmon resonance (LSPR) when irradiated at their plasmon resonance frequency, resulting in collective electron oscillations and intense local electric fields that drive the EM enhancement mechanism of SERS. Notably, AgNPs on graphene produce stronger SERS signals than those on insulating substrates. This enhancement arises from the localized surface plasmon (LSP)-induced oscillating dipole and electric field on the graphene surface (Figure 8d). Because of the two-dimensional confinement of LSP on graphene, electromagnetic waves are compressed into a smaller spatial region, leading to a reduced LSP wavelength and an enhanced reflected electromagnetic field, which further boosts the SERS signal.^69,70^

Together, these synergistic effects—enhanced light trapping from pyramidal microstructures, π–π-mediated molecular adsorption and fluorescence quenching by graphene, efficient ground-state charge transfer, and strong electromagnetic amplification from AgNPs—collectively contribute to the superior SERS performance of the AgNPs/Gr­(I)/SiO_2_/Pym Si substrate.

## 4 Conclusion

In this study, we developed a reliable CVD approach for synthesizing transfer-free conformal graphene coatings on microstructured surfaces. Such graphene-coated surfaces are promising for diverse applications, including SERS detection. The transfer-free graphene synthesized in this study exhibited high uniformity (monolayer content ∼95%), low defect density, and excellent surface conformity, even across high-curvature features, such as the apexes of silicon micropyramids. Using these conformal graphene-coated pyramidal substrates [Gr­(I)/SiO_2_/Pym Si], we achieved Raman detection of R6G at concentrations as low as 5.5 × 10^–6^ M, with excellent signal reproducibility and operational stability over 30 days. The notable SERS activity of the Gr­(I)/SiO_2_/Pym Si substrates was attributed to the CM enhancement mediated by graphene, along with enhanced light trapping and increased specific surface area resulting from the micropyramidal surface. To further boost the SERS sensitivity, AgNPs with an optimal diameter of 25.2 nm were deposited on the conformal graphene layer, producing hybrid AgNPs/Gr­(I)/SiO_2_/Pym Si substrates. These hybrid substrates exhibited a substantially higher AEF (1.08 × 10^5^) than did the Gr­(I)/SiO_2_/Pym Si substrates (45.5), enabling highly reproducible and quantitative detection of R6G down to 5.5 × 10^–9^ M. Although the EM enhancement caused by AgNPs played a dominant role in signal amplification, graphene contributed critically by suppressing the intrinsic fluorescence of silver and facilitating R6G adsorption via π–π interactions. These synergistic effects significantly improved the SERS performance of AgNPs/Gr­(I)/SiO_2_/Pym Si substrates compared with that of AgNP-only systems (AgNPs/SiO_2_/Pym Si substrates). However, the long-term stability of AgNPs/Gr­(I)/SiO_2_/Pym Si substrates remains limited due to the susceptibility of AgNPs to oxidation. To address this limitation, we are currently developing flexible SERS substrates on which plasmonic nanostructures are covered by continuous graphene films. We attempt to leverage graphene’s mechanical flexibility, chemical stability, and gas impermeability to enhance the durability of fabricated SERS substrates, which can also enable convenient analyte sampling from nonplanar or irregular surfaces via wrapping or swabbing.

## Supplementary Material



## Data Availability

Additional data, including Figures S1–S21 and Tables S1–S2, are available free of charge at http://pubs.acs.org/.

## Notes

Supporting Information provides details regarding the CVD synthesis of transfer-free graphene, the laminar flow-assisted metal etching process for preparing conformal graphene on textured substrates, sample characterization, and the AEF values at specific Raman peaks for various SERS substrates (Figures S1–S21 and Tables S1–S2) (PDF)
