# Supplementary material for: Transfer-Free Conformal Graphene Coating on Pyramidal Microstructures Decorated with Silver Nanoparticles for Superior Raman Signal Enhancement
Source: ACS Appl Mater Interfaces. 2025 Sep 12;17(38):54214–29. doi: 10.1021/acsami.5c11957 (PMC12464906; doi:10.1021/acsami.5c11957)
Supplement: Supplementary file 1 [file am5c11957_si_001.pdf]

Supporting Information

Transfer-Free Conformal Graphene Coating on Pyramidal Microstructures Decorated with Silver Nanoparticles for Superior Raman Signal Enhancement

Cheuk Yui Lai,[§] Yu-Xuan Lin,[§] En-Jing Lin, Ching-Chih Lin, and Chiao-Chen Chen[*]

Department of Chemistry
National Cheng Kung University
No.1, University Road
Tainan City 701, Taiwan

[§]These authors contributed equally to this work.
[*]Author to whom correspondence should be addressed.

E-mail: chiaochen@mail.ncku.edu.tw; Phone: +886 6 275-7575 ext.65354; Fax: +886 6 274-0552

Section 1: Chemical Vapor Deposition Synthesis of Transfer-Free Graphene

Uniform monolayer graphene can be synthesized at the interface between a catalytic metal film and a dielectric substrate through chemical vapor deposition (CVD) in a customized reactor.[S1] This study used a reactor containing a quartz slit with dimensions of 85 × 13 × 0.55 mm^3 (length × width × height) for graphene synthesis. The catalytic substrates used in this study were prepared by sputtering a 950-nm-thick Cu film onto 0.75 × 0.75-cm^2 silicon substrates, which exhibited micropyramidal surface structures covered by a 300-nm-thick SiO_2 layer. For graphene synthesis, the Cu-coated substrates were placed in the quartz slit located in a tubular reaction chamber, which was then evacuated to a pressure of ~3 × 10^{-2} Torr for 10 min. Subsequently, 200 sccm of Ar (99.9995%) and 10 sccm of H_2 (99.9995%) were introduced into the reaction chamber to increase the chamber pressure to 90 Torr. This pressure was maintained throughout the synthesis process to effectively suppress the Cu sublimation for stable graphene growth conditions, as discussed in our previous study.[S1] A tubular furnace was used to increase the chamber temperature to 950 °C within 40 min and then maintained for 30 min to anneal the substrate. Subsequently, graphene growth was initiated by introducing 1 sccm of CH_4 (99.9995%) into the reaction flow (200 sccm Ar and 10 sccm H_2), and the reaction temperature was maintained at 950 °C for 15 min. Upon the completion of graphene growth, the H_2 and CH_4 flows were terminated, and the quartz slit reactor was rapidly withdrawn from the furnace's heating zone. The system was then allowed to cool to room temperature, following which the prepared graphene-coated samples were retrieved.

Although the general framework of the CVD process for synthesizing transfer-free graphene on micropyramidal Si substrates follows our previous approach for flat Si substrates, several key parameters were re-evaluated and optimized to account for the unique challenges presented by the textured surface. Based on our experimental observations and previous literature,[S2] we hypothesized that the presence of a SiO_2 buffer layer is essential to enable uniform graphene growth on Cu-coated Si substrates. Since the formation of micropyramidal structures via alkaline etching requires a bare Si surface, an additional wet oxidation step was introduced post-texturing to grow a 300 nm-thick SiO_2 layer. When using Cu-coated micropyramidal Si substrates with the SiO_2 layer for graphene synthesis, the produced Gr(O)/Cu/Gr(I)/SiO_2/Pym Si substrate maintained good Cu coverage (Figure S5a), and successful graphene formation was confirmed by Raman measurements (Figure S5b). By contrast, without the SiO_2 layer, the CVD product exhibited clear degradation, as observed by the naked eye (Figure S1a) and SEM (Figure S1b–d). Moreover, porous features on the Si pyramids suggested structural damage (Figure S1c). The absence of metallic luster and EDS mapping (Figure S1e–i) indicated severe Cu dewetting and oxidation. While no notable Cu-Si alloy formation was detected, the data collectively confirmed that the SiO_2 buffer layer is crucial for producing conformal graphene coatings on pyramidal substrates.

Furthermore, Cu film thickness was optimized to balance between film continuity and graphene quality. As reported previously,[S1] Cu films thinner than 400 nm tend to dewet, while thicker films (≥1000 nm) promote multilayer graphene growth. A 700 nm Cu film, optimal for flat substrates, proved insufficient for textured surfaces, with severe Cu sublimation observed (Figure S2a–c), likely

due to enhanced dewetting on the rough surface. Increasing the Cu thickness to 850 nm improved film integrity, but sublimation at the pyramid apices persisted (Figure S2d–f). A final thickness of 950 nm provided the best results, with minimal Cu loss and complete coverage (Figure S2g–i).

Finally, CVD process parameters, including reaction temperature, annealing time, and growth duration were optimized. As shown in the process flowchart (Figure S3a), the CVD cycle consists of four stages: (I) temperature ramp-up, (II) substrate annealing, (III) graphene growth, and (IV) cooling. The gas compositions used were consistent with our previous studies. Reaction temperatures ≥ 900 °C were investigated, as lower temperatures yielded poor-quality graphene. Raman analysis (Figure S3b) showed that 950 °C produced graphene with the lowest thickness (I_{2D}/I_G ratio of 1.76 ± 0.35) and an acceptable defect density (I_D/I_G ratio of 0.29 ± 0.14). Subsequently, annealing and growth durations were optimized to 30 and 15 min, respectively, based on the highest I_{2D}/I_G and lowest I_D/I_G values (Figure S3c,d).

In summary, optimal parameters for CVD synthesis of transfer-free graphene on micropyramidal Si substrates include a Cu film thickness of 950 nm, a reaction temperature of 950 °C, a 30 min annealing period, and a 15 min graphene growth duration.

Section 2: Laminar Flow-Assisted Metal Etching for Preparing Conformal Transfer-Free Graphene Coatings on Textured Substrates

Following CVD synthesis, graphene films were formed on the upper Cu surface and at the Cu–SiO_2/Si interface of the pyramid-textured silicon substrate. To expose the transfer-free graphene grown at the Cu–SiO_2/Si interface, the Cu film and the graphene layer grown on it had to be

completely removed. Incomplete removal of the upper graphene layer before Cu etching would result in contamination of the interfacial graphene by the upper graphene fragments, thereby compromising the quality of the produced transfer-free graphene. To address this problem, the as-synthesized Gr(O)/Cu/Gr(I)/SiO_2/Pym Si sample (Figure 1) was immersed in 0.05 M ammonium persulfate (Alfa Aesar, 98.0%) for 5 min to etch away the outermost Cu surface and the graphene grown on it. This sample was then subjected to ultrasonication in deionized water for 10 min to ensure complete detachment of the upper graphene layer [Gr(O)]. This step resulted in the formation of a pyramid-textured SiO_2/Si substrate coated with a Cu-covered interfacial graphene layer. To remove the remaining Cu film without damaging the interfacial graphene, we employed a laminar flow-assisted etching previously reported by our group.[S3] This method utilizes a microfluidic system in which a laminar flow is established, enabling smooth and well-controlled metal etching. By avoiding turbulent flow and mechanical agitation, this approach significantly reduces the risk of introducing structural defects into the graphene film. Consequently, we successfully obtained pyramid-textured SiO_2/Si substrates uniformly coated with high-quality, transfer-free conformal graphene films.

Section 3: Sample Characterization

The surface morphologies of the prepared pyramid-textured SiO_2/Si substrates and various materials deposited on them, including sputter-deposited Cu films, transfer-free conformal graphene coatings, and AgNPs, were characterized through field-emission scanning electron microscopy (SEM; HITACHI SU8000 and Zeiss AURIGA Compact FIB-SEM). Dimensional analysis of the pyramidal microstructures and AgNPs was performed using ImageJ software.[S4] To verify the surface

functionalization of SiO_2/Pym Si substrates with graphene and AgNPs, an X-ray photoelectron spectrometer (ULVAC-PHI VersaProbe 4) was utilized to examine the chemical compositions of these substrates.

Section 4: Focus Optimization Using FocusTrack Mode

To minimize focus-related variations during Raman measurements, we employed the FocusTrack mode of the Renishaw inVia Reflex Raman microscope. FocusTrack is a software-based autofocus system that adjusts the Z-position of the sample stage based on surface reflections before each spectrum acquisition. This mode dynamically maintains optical focus by compensating for surface height differences, such as those introduced by our micropyramidal SERS substrates.

In our quantitative experiments, analytes at five or six different concentrations were deposited as an array on a single substrate using a fixed-volume drop-drying method. All measurements were performed under identical laser power, exposure time, and object lens settings to ensure consistency. Each sample spot was mapped over a 5×4 grid (20 points total) to collect Raman spectra for statistical analysis. Since FocusTrack mode reoptimizes the focus at every measurement point, rather than relying on a fixed focal plane, it effectively mitigates the impact of surface irregularities and local height variations.

Section 5: Calculation of Relative Standard Deviation (RSD)

To evaluate the reproducibility and uniformity of SERS signals, the relative standard deviation (RSD) was calculated from a series of repetitive Raman measurements taken from different positions on a substrate. The RSD is defined as the ratio of the standard deviation to the mean of the

measurements, expressed as a percentage. It quantifies the relative variability of the data and is widely used to assess measurement precision. The calculation involves determining the mean intensity (μ) and the absolute standard deviation (σ). The mean intensity of a selected Raman peak (e.g., the R6G peak at 613 cm^{-1}) across multiple measurements is calculated using eq S1, where μ is the mean intensity, x_i is the numerical value of the ith Raman intensity measurement, and N is the total number of measurements.

$$\mu = \frac{1}{N}\sum_{i=1}^{N} x_i \tag{eq S1}$$

$$\sigma = \sqrt{\frac{1}{N-1}\sum_{i=1}^{N}(x_i - \mu)^2} \tag{eq S2}$$

$$RSD(\%) = \left(\frac{\sigma}{\mu}\right) \times 100 \tag{eq S3}$$

The absolute standard deviation (σ), which measures the spread of the data around the mean, is calculated using eq S2. The RSD is then obtained by normalizing the absolute standard deviation (σ) to the mean (μ) and expressing the result as a percentage, as shown in eq S3. This method was applied to the Raman intensity values extracted from the characteristic peaks of R6G in both the microscale 100-point and macroscale 9-point mappings to evaluate the spatial uniformity of the SERS signal across the substrate surface.

Section 6: Fermi Level Shift in the AgNPs/Graphene Hybrid System

Graphene exhibits charge neutrality with a Fermi level (~ −4.6 eV) at the Dirac point. However, when graphene contacts metallic substrates such as Ag (work function, W_{Ag} ~ 4.92 eV), its electronic properties are modified (Figure S21b). While a simple work function comparison between graphene and Ag suggests the hole doping of graphene ($W_G < W_{Ag}$, Figure S21c, left image), both theoretical

and experimental studies report n-type doping when Ag contacts graphene, implying that electrons are injected from Ag to graphene.[S5-7]

This phenomenon arises from the charge redistribution at the graphene-metal interface. This interfacial charge redistribution is not only a result of the electron transfer between the metal and graphene according to their work functions, but also a contribution from a graphene-metal chemical interaction, which induces a potential shift (ΔV) shown in Figure S21b.[S7] At the equilibrium graphene-metal separation (d ~ 3.3 Å), where the attractive and repulsive forces between metal and graphene are balanced for the most stable metal/graphene configuration, ΔV significantly alters the hybrid system's work function (W), making simple work function comparisons insufficient to predict the doping behavior of graphene.

As a result, Ag deposition raises the Fermi level of graphene to ~ −4.40 eV (−4.24 ~ −4.56 eV reported in literature), indicating n-type doping of graphene.[S5, 7] In our system, the Fermi levels (E_F) of graphene-based substrates are illustrated in Figure S21a for Gr(I)/SiO_2/Pym Si and Figure S21c (right image) for AgNPs/Gr(I)/SiO_2/Pym Si, along with the energy levels of R6G (HOMO at −5.7 eV and LUMO at −3.4 eV).

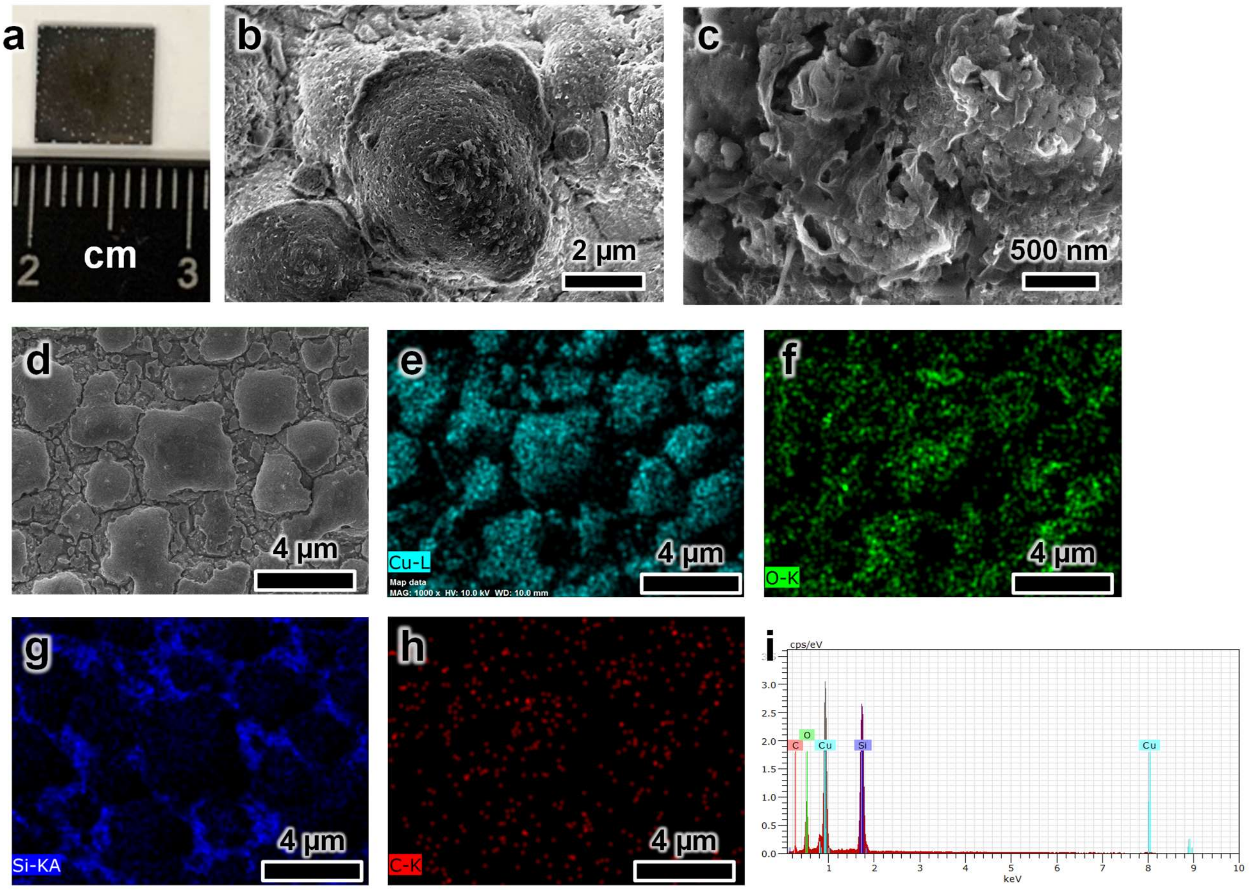

Figure S1. (a) Photograph and (b–d) SEM images at different magnifications of the CVD product prepared with a Cu-coated micropyramidal Si substrate without the SiO_2 buffer layer. (e–f) EDS mappings of the area shown in (d) for revealing the distribution of (e) Cu, (f) O, (g) Si, and (h) C. (i) EDS spectra determined from the sample area shown in (d).

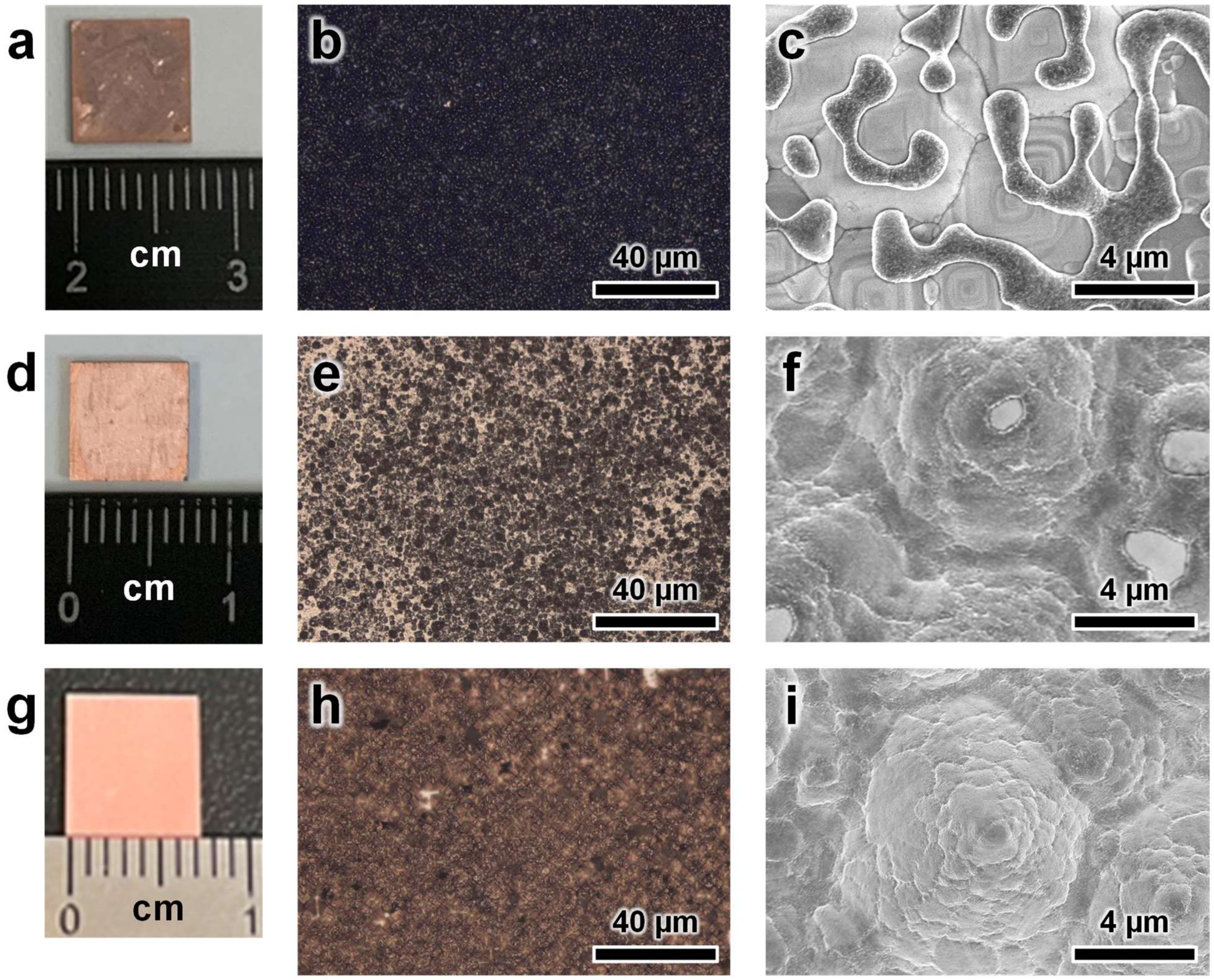

Figure S2. (a, d, g) Photograph, (b, e, h) optical micrographs, and (c, f, i) SEM images of Gr(O)/Cu/Gr(I)/SiO_2/Pym Si substrates synthesized by using Cu films with different thicknesses: (a–c) 700 nm, (d–f) 850 nm, and (g–i) 950 nm.

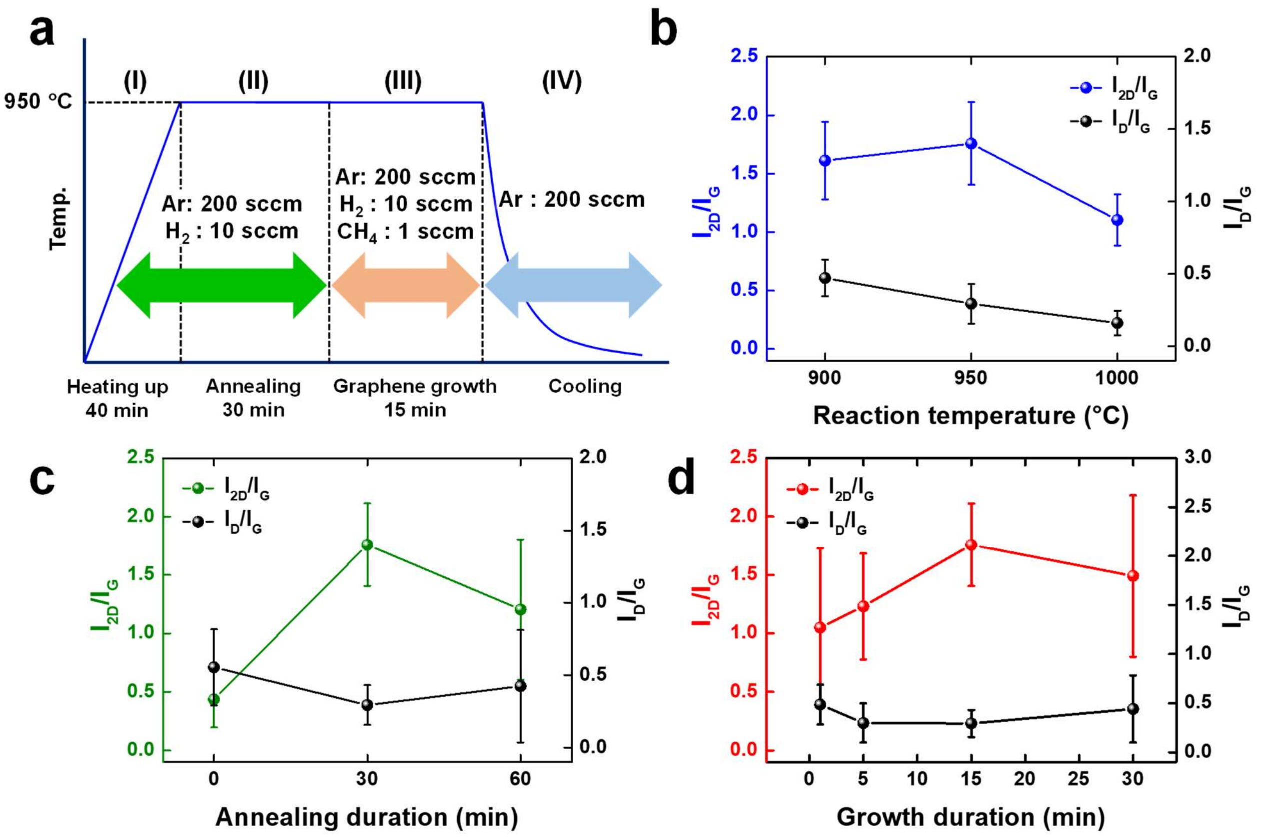

Figure S3. (a) Flowchart of the CVD process used to synthesize Gr(O)/Cu/Gr(I)/SiO_2/Pym Si substrates. Influence of CVD parameters, including the (b) reaction temperature, (c) annealing duration, and (d) growth duration, on the quality of the produced graphene was investigated systematically in terms of the I_{2D}/I_G and I_D/I_G ratios determined from Raman measurements.

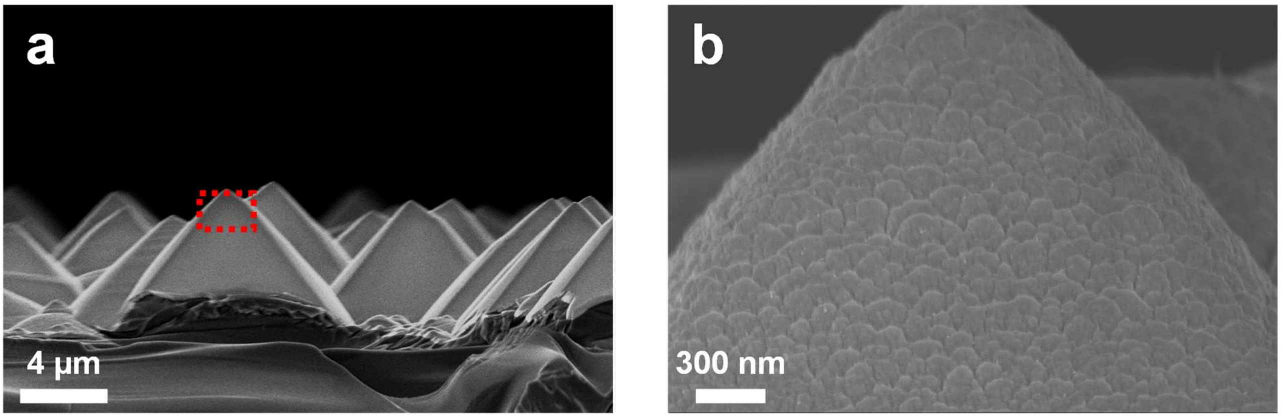

Figure S4. (a) Cross-sectional SEM image of micropyramidal structures on a textured silicon substrate coated with a Cu film through ion-beam sputtering. (b) High-magnification SEM image of the region marked by the red rectangle in (a). This image indicates that the Cu film consisted of densely packed Cu nanoparticles.

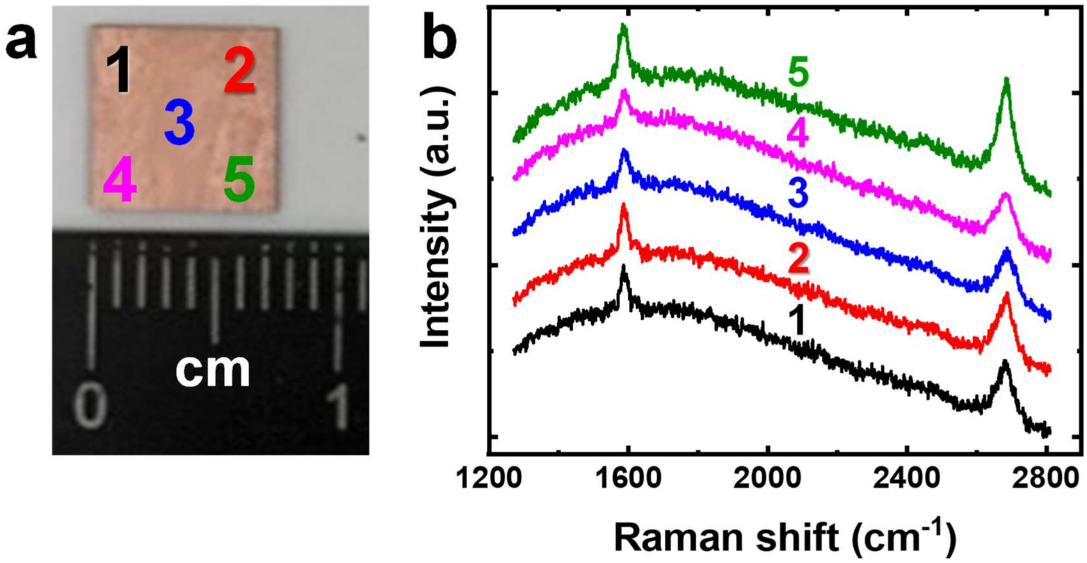

Figure S5. (a) Photograph of an as-synthesized Gr(O)/Cu/Gr(I)/SiO_2/Pym Si substrate. (b) Raman spectra acquired for the five positions indicated in (a). Although photoluminescence originating from the Cu film caused interference in the Raman spectra, the presence of graphene on the upper Cu surface was confirmed by the characteristic G and 2D bands at ~1585 and ~2696 cm^{-1}, respectively.

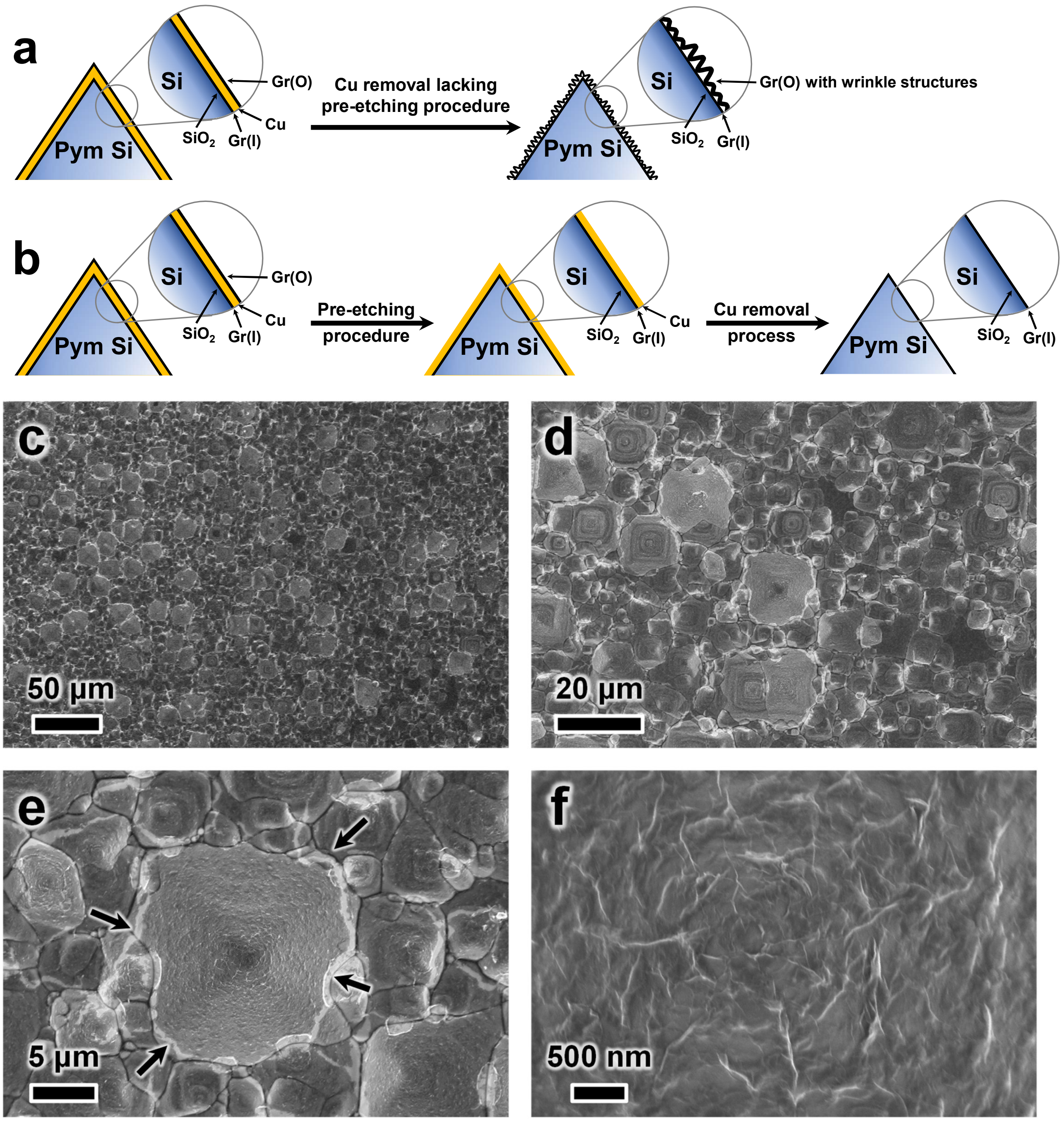

Figure S6. The Cu removal processes (a) lacking a thorough pre-etching procedure for preparing Gr(O)/Gr(I)/SiO_2/Pym Si and (b) containing a thorough pre-etching procedure for preparing Gr(I)/SiO2/Pym Si, respectively. (c–f) Top-view SEM images of a representative Gr(O)/Gr(I)/SiO_2/Pym Si substrate recorded at different magnifications.

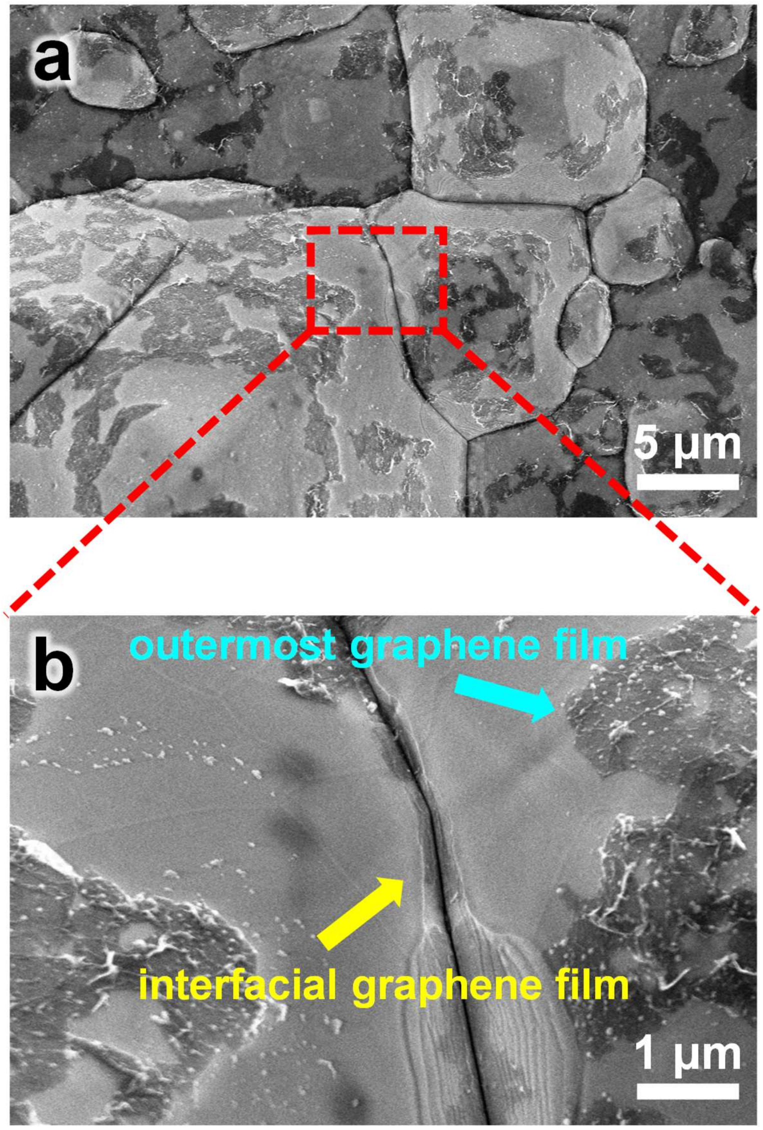

Figure S7. SEM images of transfer-free graphene films grown on a pyramid-textured surface subjected to incomplete pre-etching before Cu removal. (a) Thick graphene flakes, originally grown on the upper Cu surface, were deposited on the underlying substrate and appeared as dark fragments scattered over the pyramidal structures. (b) High-magnification image revealing a smooth, conformal thin film covering the textured surface, implying the existence of an interfacial graphene at the Cu–SiO_2 interface.

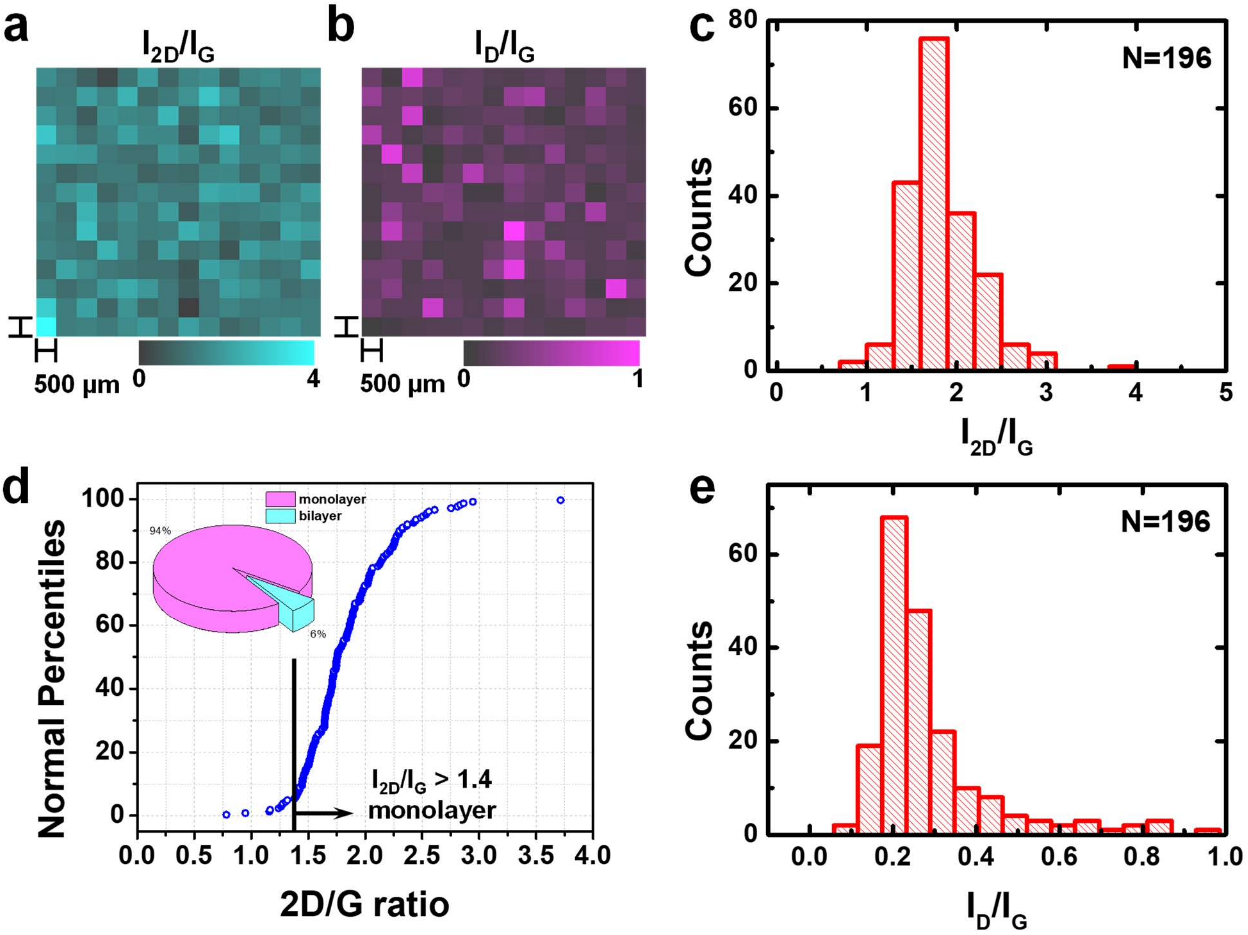

Figure S8. Two-dimensional Raman maps of the (a) I_{2D}/I_G and (b) I_D/I_G ratios obtained over a macroscale area of 0.7 × 0.7 cm^2 with a spatial resolution of 14 × 14 pixels (0.5 mm/pixel) on a Gr(I)/SiO_2/Pym Si substrate with an overall size of 0.75 × 0.75 cm^2. (c, e) Histograms showing the statistical distributions of the (c) I_{2D}/I_G and (e) I_D/I_G ratios corresponding to the data in (a) and (b), respectively. (d) Probability plot of the I_{2D}/I_G ratios derived from the Raman map shown in (a).

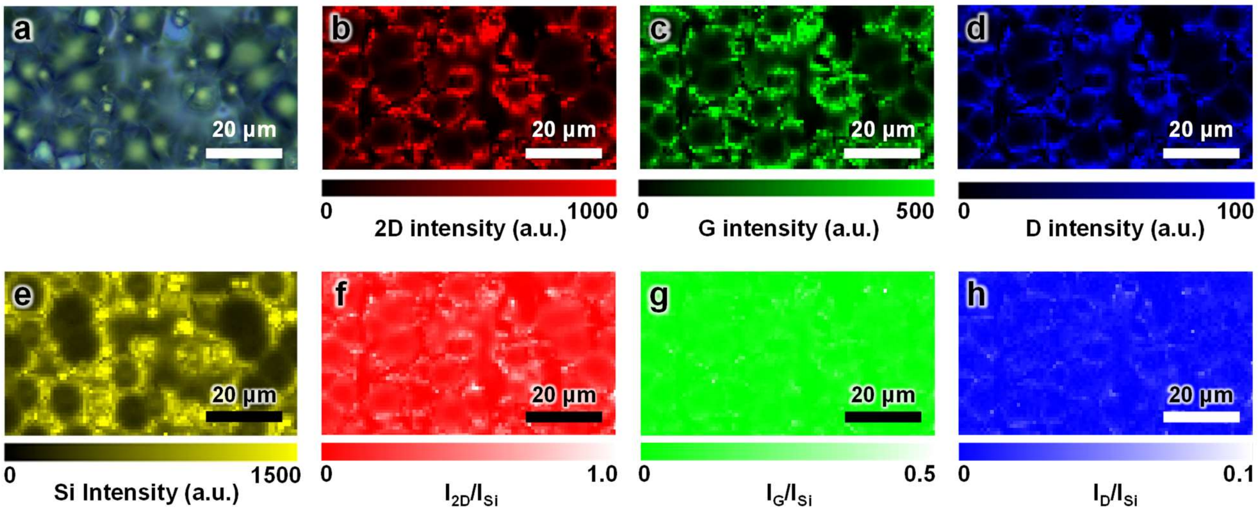

Figure S9. (a) Optical micrograph captured by focusing on the basal joints between pyramidal microstructures in the region selected for two-dimensional Raman mapping. (b–e) Raman intensity maps for the (b) 2D, (c) G, and (d) D bands of graphene and for the (e) Si peak at 520.5 cm^{-1}. Spatial variations in intensity across these maps reflect the topographical height differences of the underlying pyramid structures, which influence the observed Raman signal and obscure certain regions of graphene coverage. (f–h) Raman maps of the (f) I_{2D}/I_{Si}, (g) I_G/I_{Si}, and (h) I_D/I_{Si} ratios. These maps confirmed the uniform and continuous coverage of transfer-free graphene across the pyramid-textured substrate.

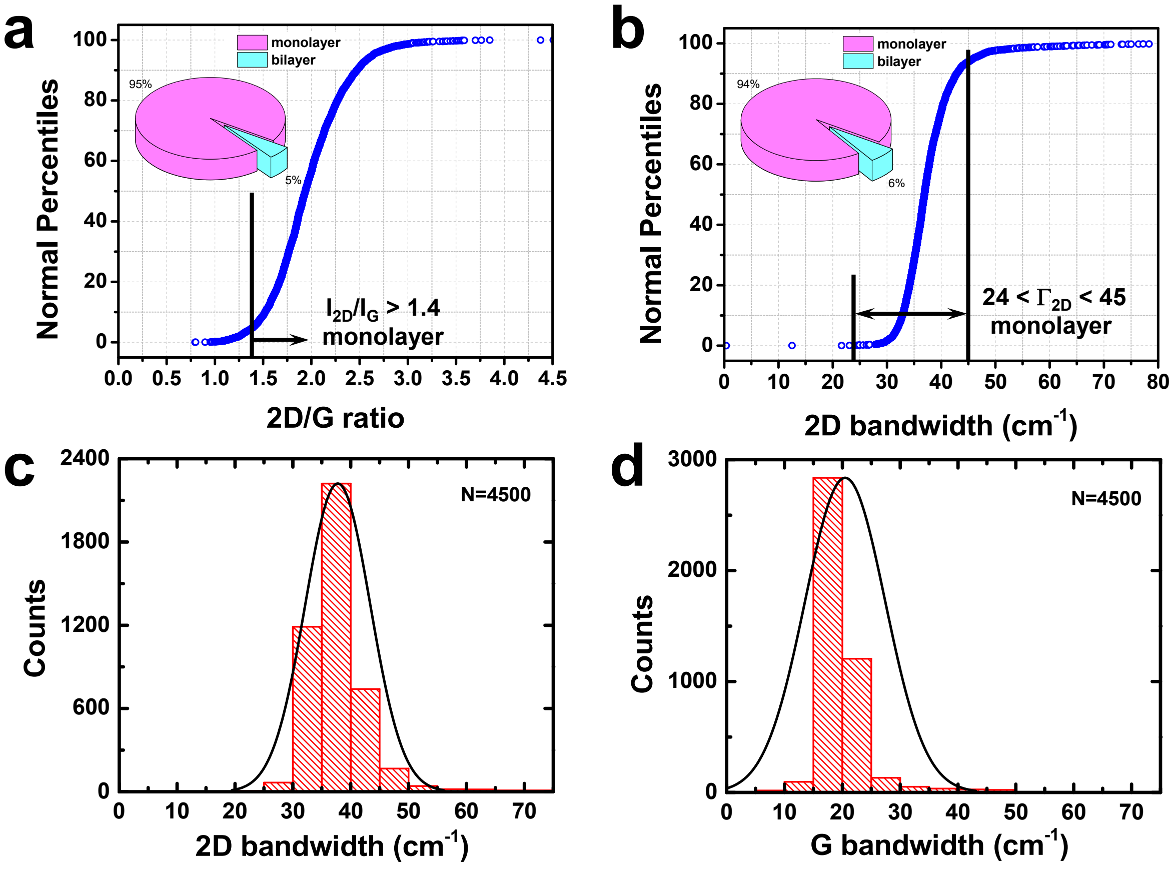

Figure S10. (a, b) Probability plots of (a) I_{2D}/I_G ratios and (b) 2D bandwidths (full width at half maximum) determined from Raman maps for the transfer-free conformal graphene prepared in this study. These plots suggest that the graphene had 94–95% monolayer structure. (c, d) Histograms of the bandwidths (full width at half maximum) of the (c) 2D and (d) G bands in the Raman maps.

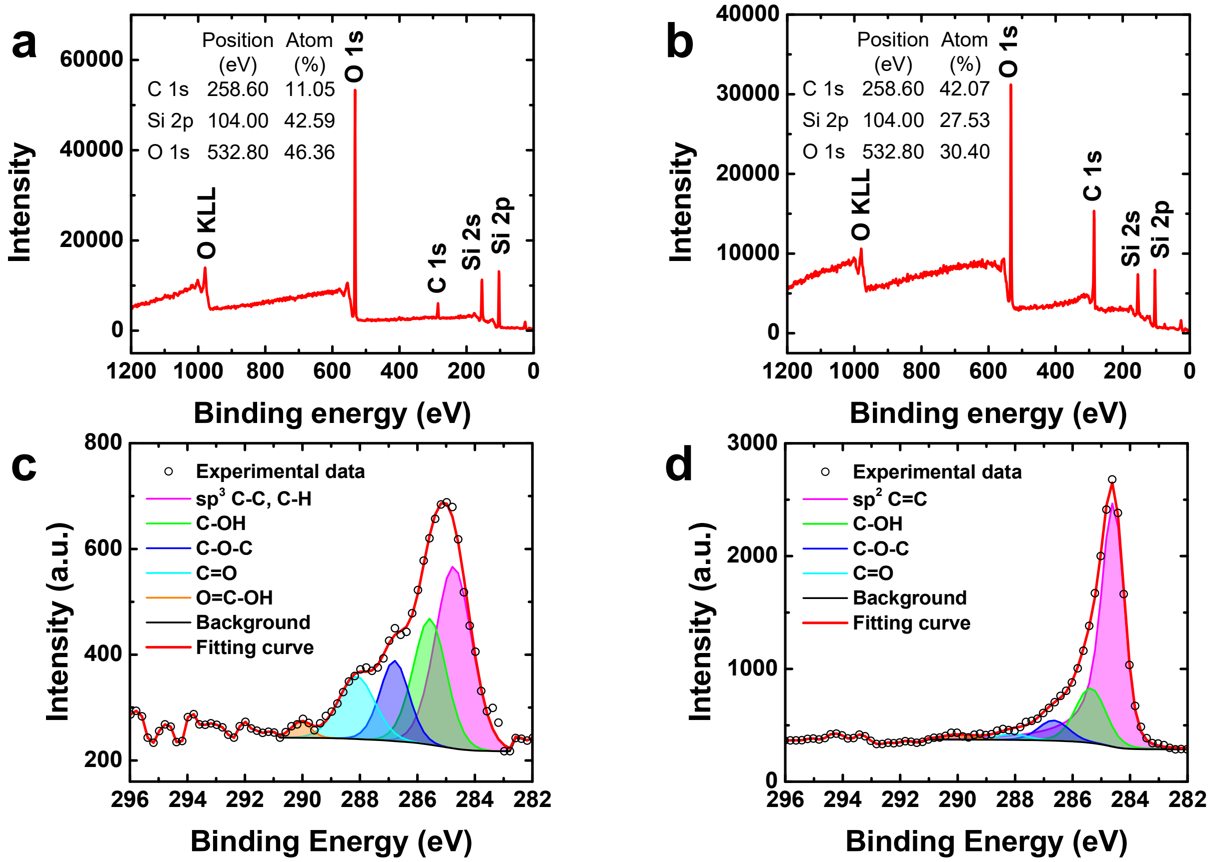

Figure S11. X-ray photoelectron spectroscopy (XPS) characterization of bare and graphene-coated SiO_2/Pym Si substrates. (a, b) XPS survey spectra of a (a) SiO_2/Pym Si and (b) Gr(I)/SiO_2/Pym Si substrate. (c, d) High-resolution C 1s spectra of the (c) SiO_2/Pym Si and (d) Gr(I)/SiO_2/Pym Si substrate. Deconvolution of the C 1s spectra revealed the chemical states of carbon species present on the surface of each substrate.

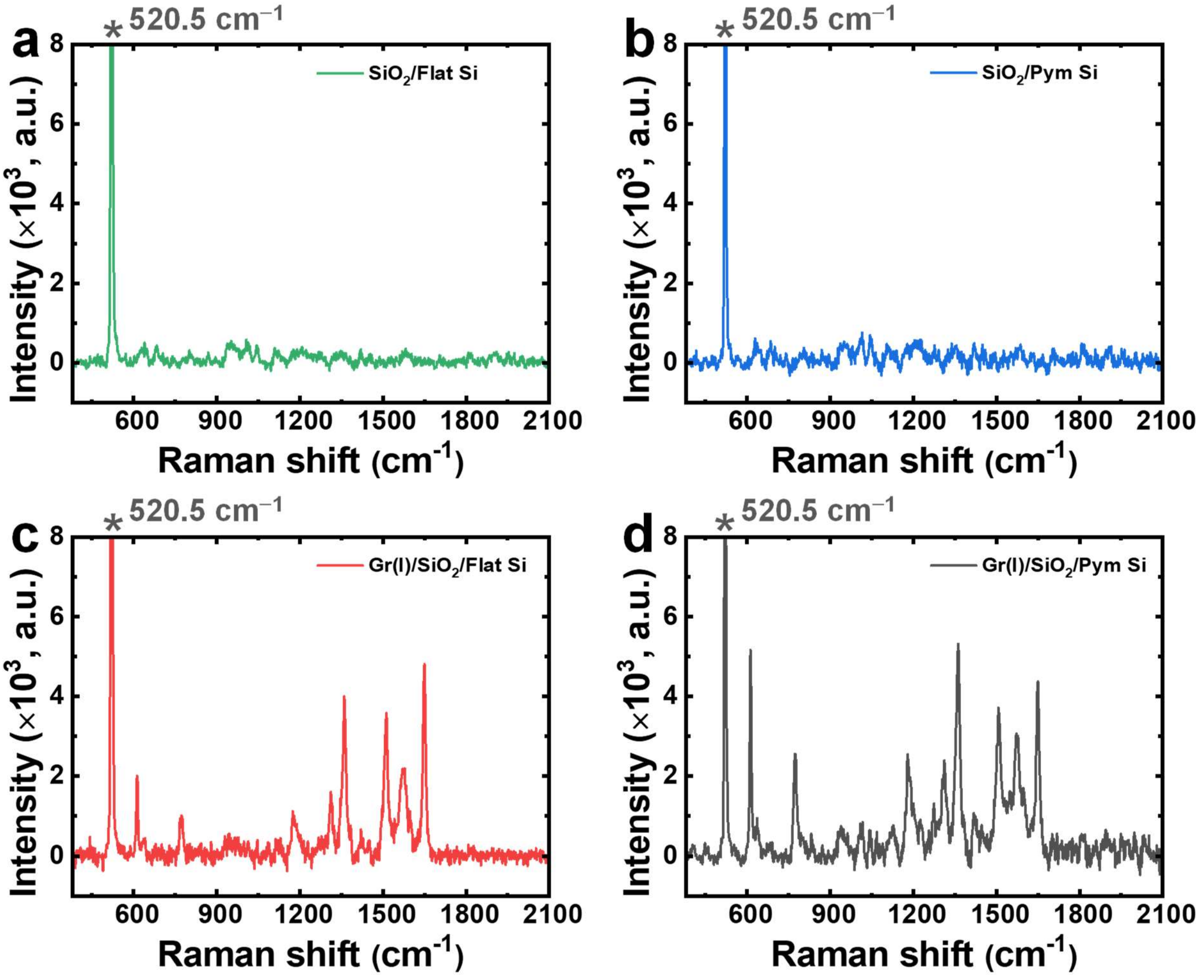

Figure S12. Raman spectra of R6G recorded on the (a) SiO_2/Flat Si, (b) SiO_2/Pym Si, (c) Gr(I)/SiO_2/Flat Si, and (d) Gr(I)/SiO_2/Pym Si substrates, respectively.

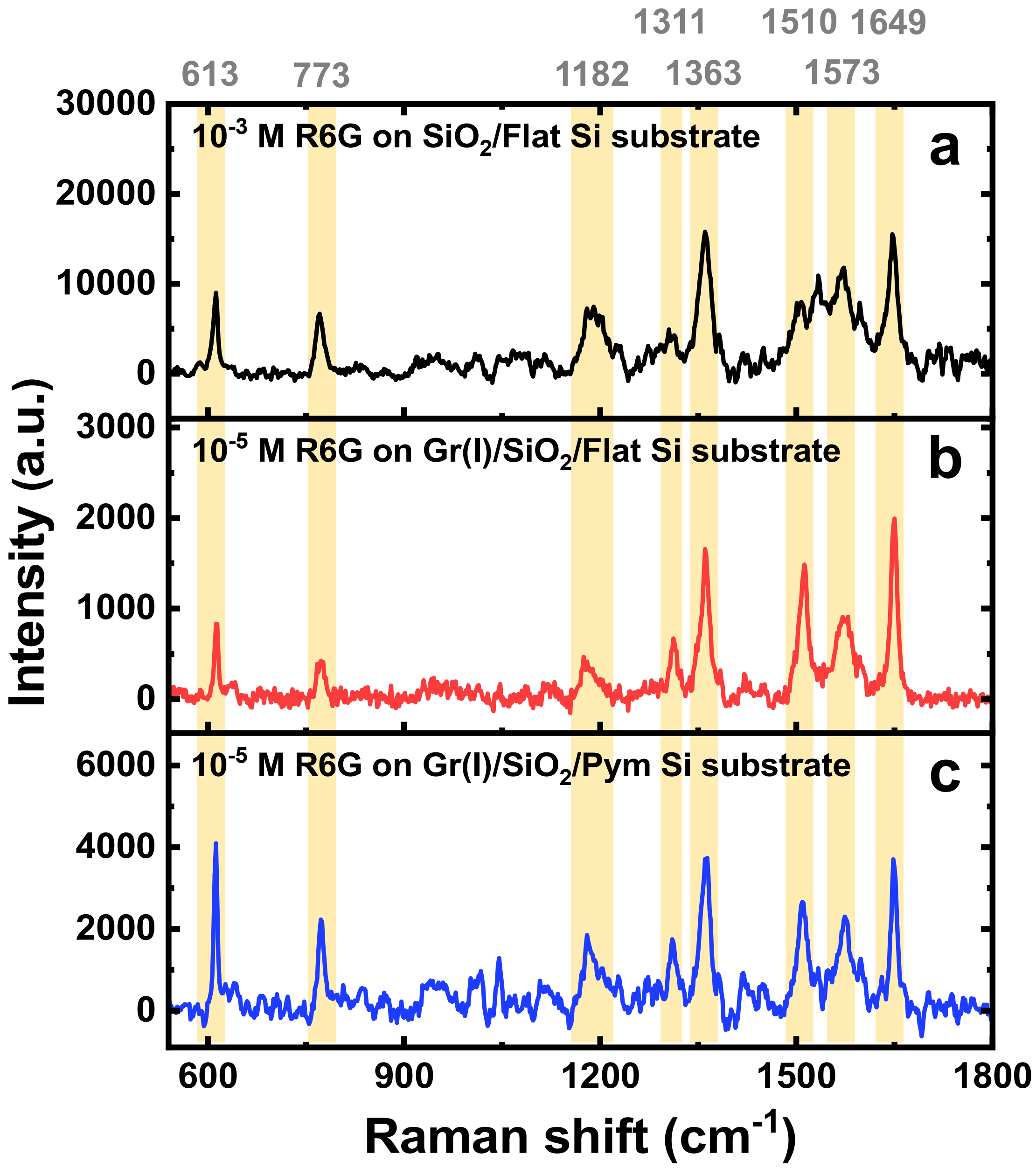

Figure S13. (a) Raman spectrum of 10^{-3} M rhodamine 6G (R6G) on a SiO_2/Flat Si substrate. (b, c) Surface-enhanced Raman scattering (SERS) spectra of 10^{-5} M R6G on (b) Gr(I)/SiO_2/Flat Si and (c) Gr(I)/SiO_2/Pym Si substrates.

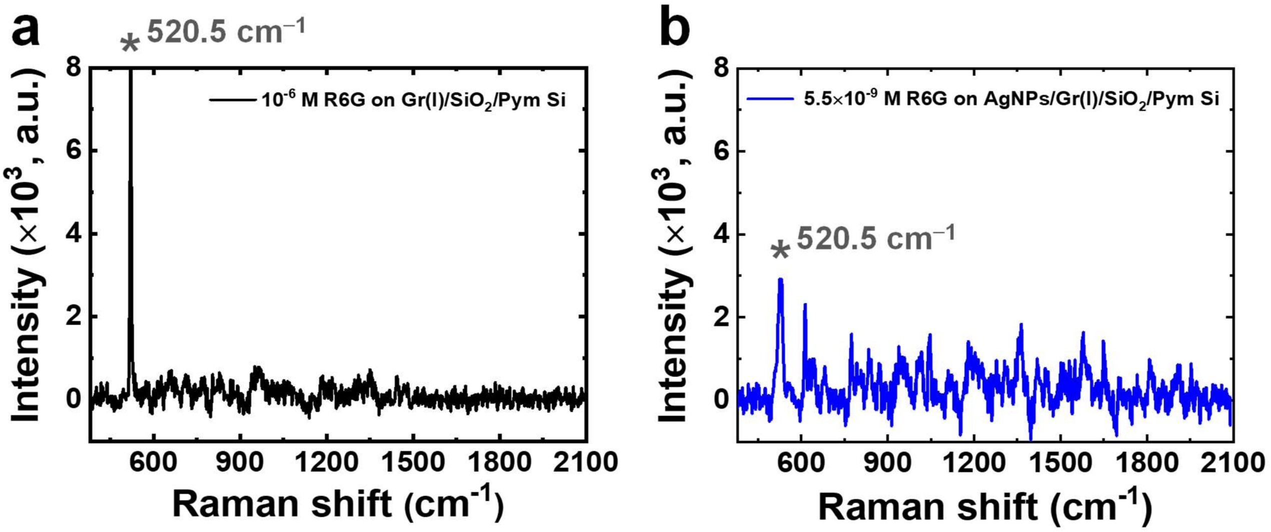

Figure S14. Raman spectra of (a) 10^{-6} M R6G on the Gr(I)/SiO_2/Pym Si substrate and (b) 5.5 × 10^{-9} M R6G on the AgNPs/Gr(I)/SiO_2/Pym Si substrate.

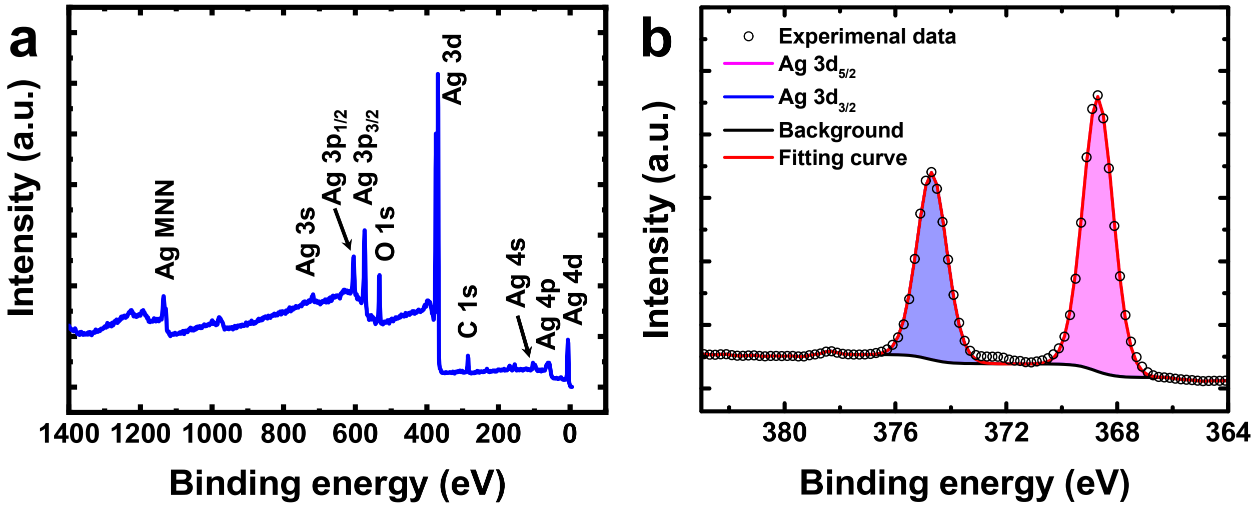

Figure S15. (a) XPS survey spectrum for the surface of a AgNPs/Gr(I)/SiO_2/Pym Si substrate. (b) High-resolution Ag 3d spectrum of the AgNPs/Gr(I)/SiO_2/Pym Si substrate, with deconvolution indicating the high purity of metallic silver and no detectable oxidation.

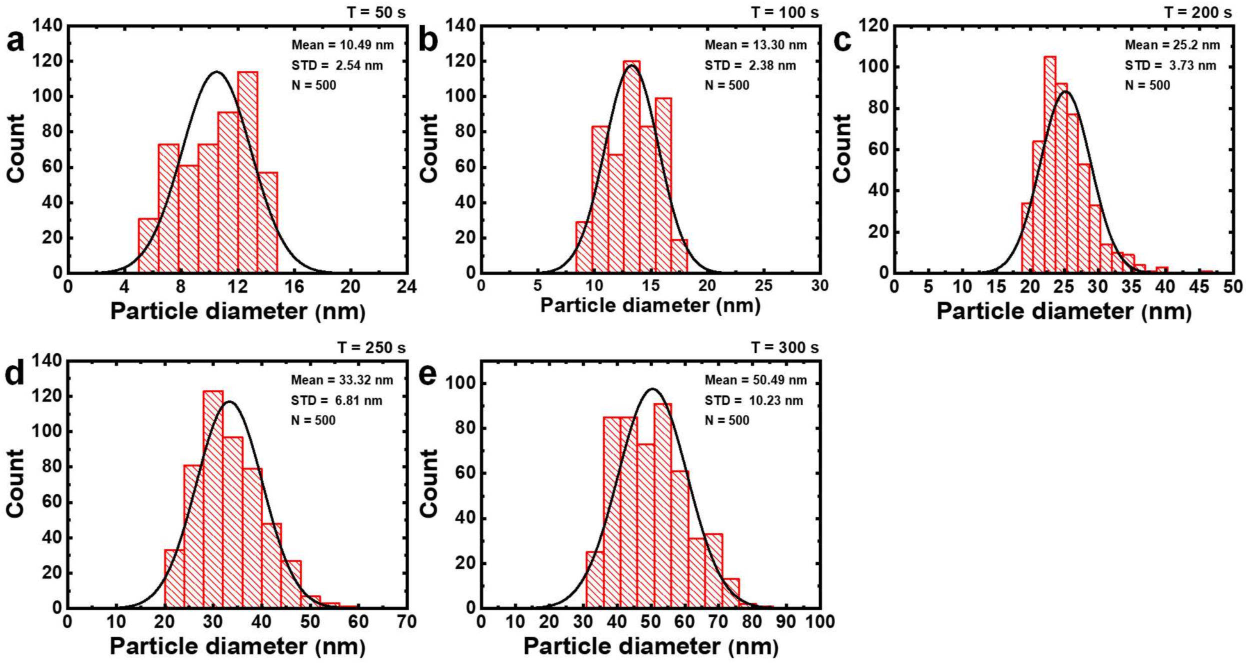

Figure S16. Histograms of the size distributions of AgNPs deposited for (a) 50, (b) 100, (c) 200, (d) 250, and (e) 300 s. These histograms were determined from the SEM images in Figure 6a–e, respectively.

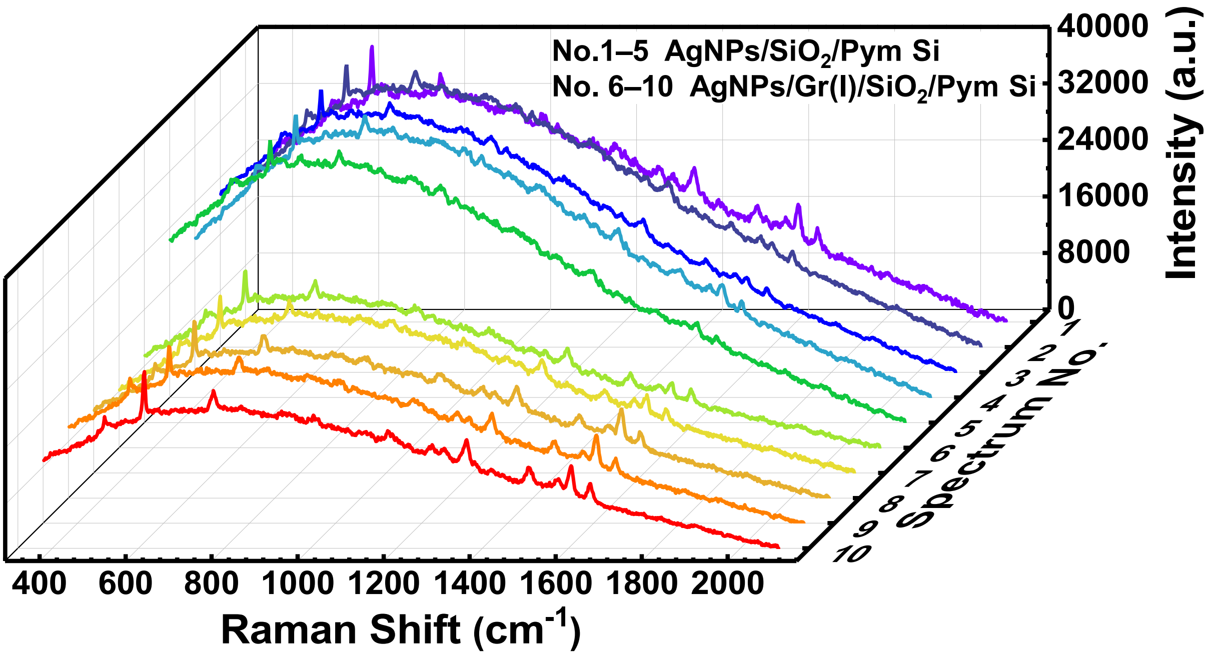

Figure S17. Raw SERS spectra (without background subtraction) of 10^{-7} M R6G on AgNPs/SiO_2/Pym Si and AgNPs/Gr(I)/SiO_2/Pym Si substrates. According to these spectra, the intrinsic silver fluorescence on the AgNPs/Gr(I)/SiO_2/Pym Si substrates was effectively suppressed, suggesting the powerful fluorescence quenching effect of graphene.

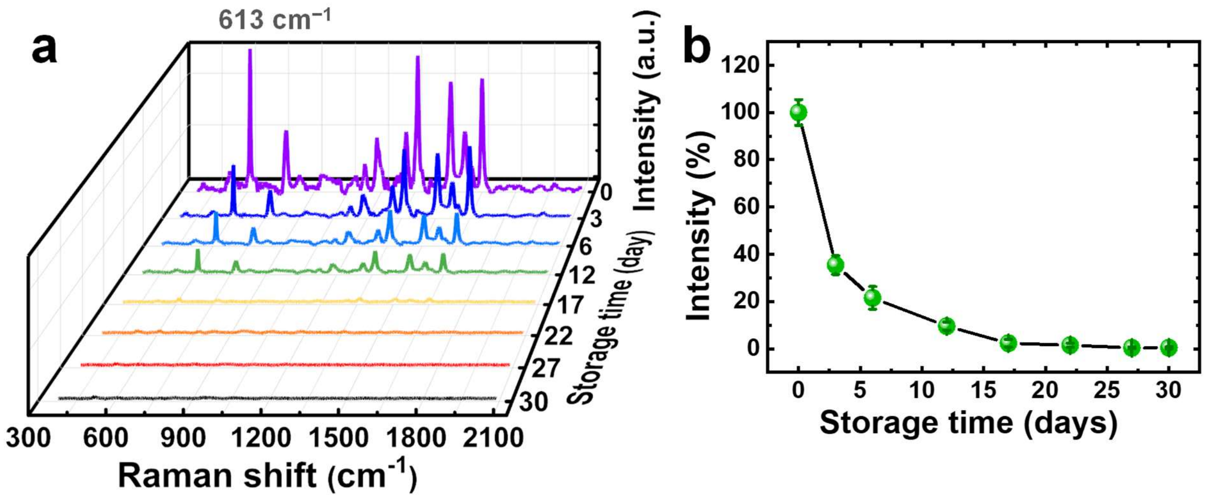

Figure S18. (a) SERS spectra recorded over 30 days for 10^{-7} M R6G on a AgNPs/Gr(I)/SiO_2/Pym Si substrate stored at 40% relative humidity. (b) Plot of variations in signal intensity at 613 cm^{-1} as a function of time, derived from the spectra shown in (a). These variations reveal the limited long-term stability of the AgNPs/Gr(I)/SiO_2/Pym Si substrate stored at 40% relative humidity.

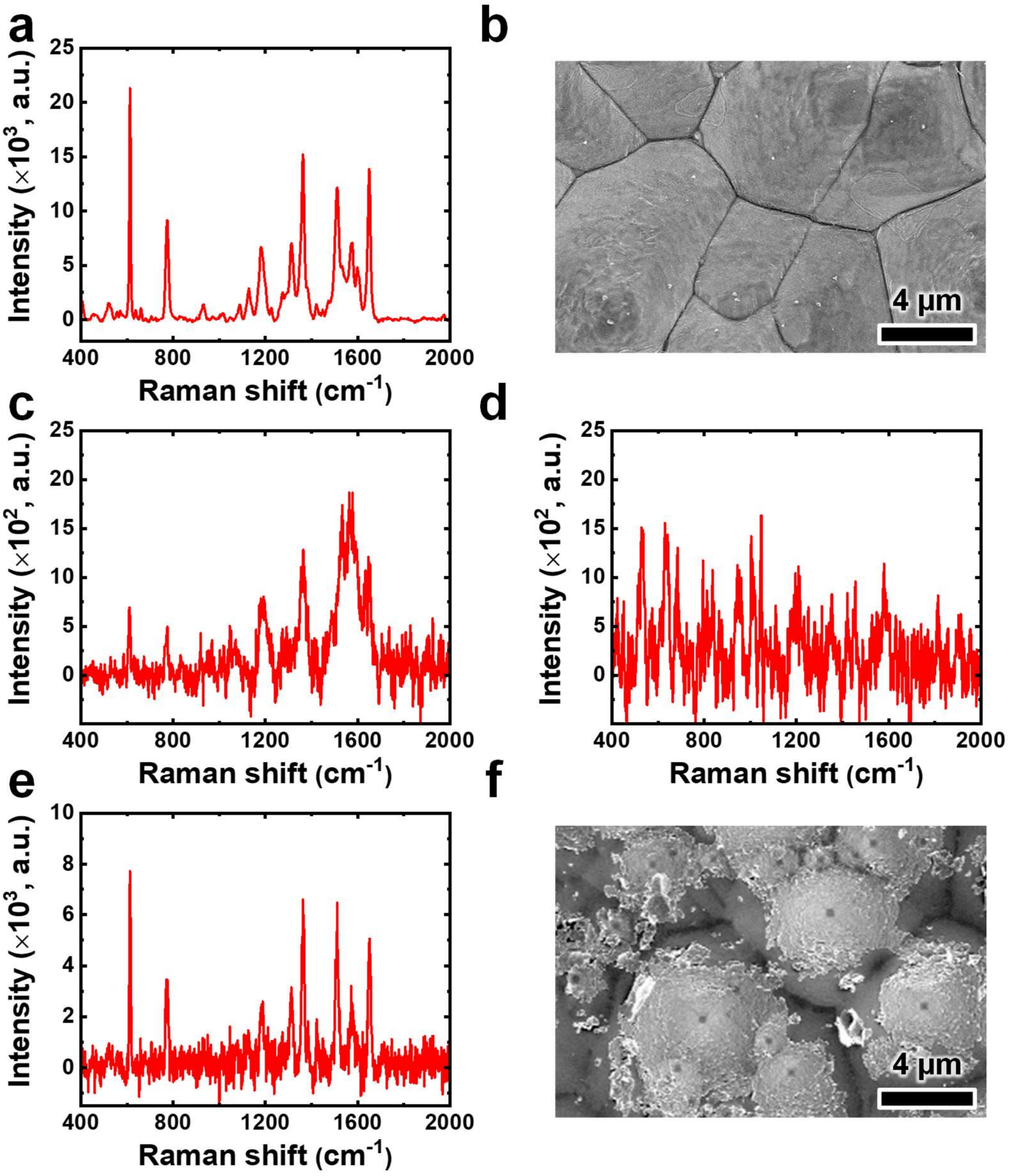

Figure S19. (a) Raman spectrum of 10^{-7} M R6G recorded on a freshly prepared AgNPs/Gr(I)/SiO_2/Pym Si with a uniform coating of AgNPs/Gr(I) film, as shown in the SEM image (b). (c) Raman spectrum recorded from the same substrate after mild cleaning with deionized water, and (d) after subsequent rigorous cleaning using acetone and 3-minute sonication. (e) Raman spectrum of 10^{-7} M R6G recorded on the reused substrate. (f) SEM image of the reused substrate, showing partial detachment of the AgNPs/Gr(I) film from the micropyramidal microstructures.

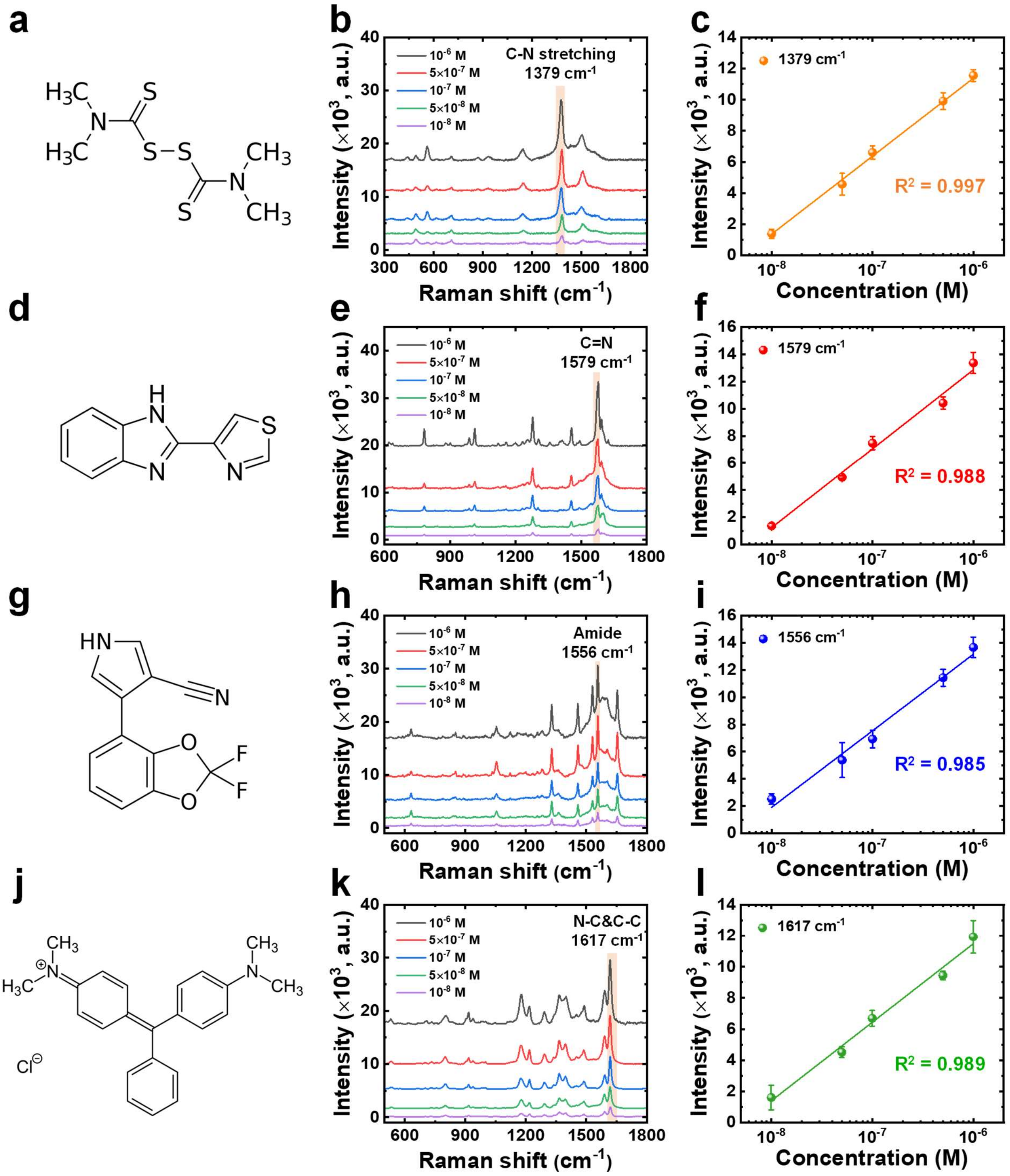

Figure S20. (a, d, g, j) Chemical structures, along with (b, e, h, k) SERS spectra at various concentrations (10^{-6}–10^{-8} M), and (c, f, i, l) corresponding calibration curves of four representative fungicides recorded on AgNPs/Gr(I) pyramid-textured substrates: (a–c) thiram, (d–f) thiabendazole, (g–i) fludioxonil, and (j–l) malachite green.

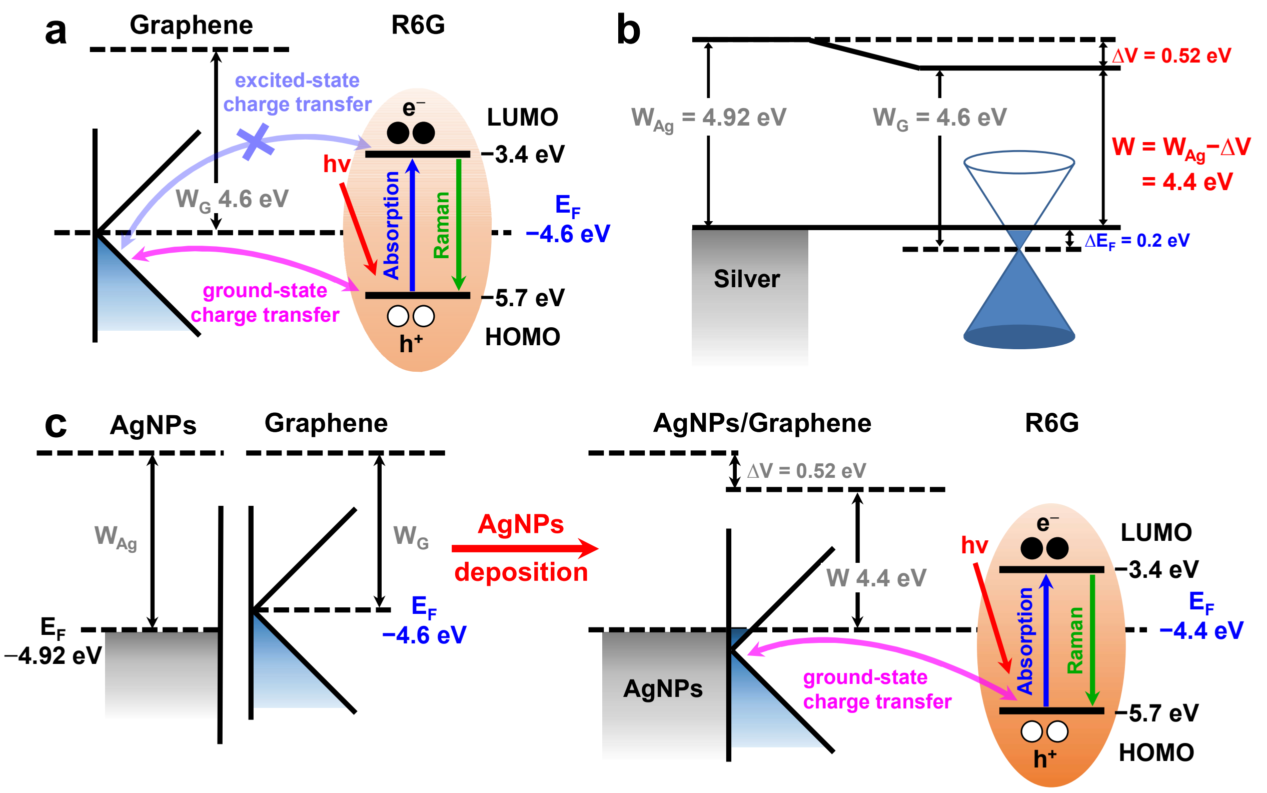

Figure S21 (a) Schematic of charge-transfer pathways between the HOMO of R6G and the Fermi level of graphene (~ −4.60 eV) on a Gr(I)/SiO_2/Pym Si substrate. (b) Schematic of the parameters and corresponding values used to calculate the potential shift (ΔV) at the graphene-silver interface, reported in *Phys. Rev. Lett.* **2018**, *101*, 0268030. (c) Band alignment after AgNP deposition on graphene, showing n-type doping of graphene to induce an upward shift of the Fermi level to ~ −4.40 eV in the AgNPs/graphene hybrid system. The corresponding ground-state charge transfer between R6G (HOMO) and the Fermi level of the AgNPs/Gr(I)/SiO_2/Pym Si substrate is also illustrated.

Table S1. Comparison of Direct CVD Synthesis for Transfer-Free Conformal Graphene Coatings on 3D (Micro)structures

**Ref.**	**Synthesis parameters**						**Figure of merit for the quality of transfer-free graphene**				
	**Category of CVD**	**Substrate type**	**Pressure (torr)**	**Carbon source**	**Growth temp. (°C)**	**Synthesis duration [a)] (min)**	**Morphology of graphene coating**	**Average I_{2D}/I_G**	**Average I_D/I_G**	**Layer Number [b)]**	**Aspect ratio of 3D structure (height/width)**
S8	metal catalytic (Cu foil)	microhemispherical Sapphire	760	CH_4	1000	45	continues film	0.85 [c)]	2.31	2 layers	3:2
S9	metal catalytic (distant Cu vapor)	inverse micropyramidal SiO_2/Si	1.53×10^{-2}	TPN [d)]	900	~ 60	continues film	0.45	0.09	few layers	2:3
S10	plasma enhanced	silicon nanoholes	0.315	CH_4	750	40	continues film	0.57	2.14	few layers	5:1
S11	plasma enhanced	micropyramidal Al_2O_3/Si	1.65×10^{-2}	CH_4	750	30	vertical nano hills	0.33	1.24	few layers	7:10
S12	plasma enhanced	micropyramidal Si	0.30	CH_4	750	55	(vertical) nanowalls	0.97	1.0	2 layers	7:10
S13	metal catalytic (Ni powder)	cm-sized, arbitrarily shaped ceramic	ambient	CH_4	1000	300	continues film	0.913 ± 0.112	0.0649 ± 0.0139	2 layers	N.A.
S14	metal catalytic (distant Ni vapor)	Si nanorod arrays	0.9	CH_4	1100	20	continues film	0.24	2	3–5 layers	4:1
**This work**	metal catalytic (deposited Cu film)	micropyramidal SiO_2/Si	90	CH_4	950	45	continues film	1.98 ± 0.65	0.21 ± 0.08	95% monolayer 5% bilayer	7:10

a) The synthesis duration includes both the annealing and growth stages required to obtain a conformal graphene coating, but excludes the initial ramp-up heating time.

b) The layer numbers are clearly stated or estimated based on the Raman measurements provided in the corresponding literature.

c) Synthesis conditions and graphene qualities that are less favorable or less competitive than those achieved in this study are shaded in gray.

d) TPN: 1,2,3,4-tetraphenylnapthalene (solid carbon source)

Table S2. Analytical Enhancement Factors Determined for Various Substrates by Using Characteristic Raman Peaks of R6G

Characteristic peak (cm^{-1})	Assignment[S15, 16]	Substrate	Signal type	Intensity (a.u.)	R6G concentration (M)	Enhancement factor
613	C–C ring in-plane bending	SiO_2/Flat Si	Raman	8995.67	10^{-3}	N.A.
		Gr(I)/SiO_2/Flat Si	SERS	832.81	10^{-5}	9.26
		Gr(I)/SiO_2/Pym Si		4096.56	10^{-5}	45.54
		AgNPs/SiO_2/Pym Si		6121.64	10^{-7}	6.81×10^{3}
		AgNPs/Gr(I)/SiO_2/Pym Si		9749.80	10^{-8}	1.08×10^{5}
773	C–H out-of-plane bending	SiO_2/Flat Si	Raman	6671.58	10^{-3}	N.A.
		Gr(I)/SiO_2/Flat Si	SERS	420.99	10^{-5}	6.31
		Gr(I)/SiO_2/Pym Si		2227.36	10^{-5}	33.39
		AgNPs/SiO_2/Pym Si		2745.95	10^{-7}	4.12×10^{3}
		AgNPs/Gr(I)/SiO_2/Pym Si		3560.22	10^{-8}	5.34×10^{4}
1182	C–H in-plane bending	SiO_2/Flat Si	Raman	7282.90	10^{-3}	N.A.
		Gr(I)/SiO_2/Flat Si	SERS	416.13	10^{-5}	5.71
		Gr(I)/SiO_2/Pym Si		1852.89	10^{-5}	25.44
		AgNPs/SiO_2/Pym Si		1399.60	10^{-7}	1.93×10^{3}
		AgNPs/Gr(I)/SiO_2/Pym Si		2202.83	10^{-8}	3.02×10^{4}
1311	hybrid vibration associated with aromatic rings and NHC_2H_5 group	SiO_2/Flat Si	Raman	4449.21	10^{-3}	N.A.
		Gr(I)/SiO_2/Flat Si	SERS	668.56	10^{-5}	15.03
		Gr(I)/SiO_2/Pym Si		1748.71	10^{-5}	39.30
		AgNPs/SiO_2/Pym Si		1036.77	10^{-7}	2.33×10^{3}
		AgNPs/Gr(I)/SiO_2/Pym Si		1955.45	10^{-8}	4.40×10^{4}
1363	aromatic C–C stretching	SiO_2/Flat Si	Raman	15785.07	10^{-3}	N.A.
		Gr(I)/SiO_2/Flat Si	SERS	1657.72	10^{-5}	10.50
		Gr(I)/SiO_2/Pym Si		374.378	10^{-5}	23.70
		AgNPs/SiO_2/Pym Si		3010.54	10^{-7}	1.91×10^{3}
		AgNPs/Gr(I)/SiO_2/Pym Si		5012.37	10^{-8}	3.18×10^{4}

Table S2. Analytical Enhancement Factors Determined for Various Substrates by Using Characteristic Raman Peaks of R6G (Continued)

Characteristic peak (cm^{-1})	Assignment[S15, 16]	Substrate	Signal type	Intensity (a.u.)	Sample concentration (M)	Enhancement factor
1510	aromatic C–C stretching	SiO_2/Flat Si	Raman	7974.06	10^{-3}	N.A.
		Gr(I)/SiO_2/Flat Si	SERS	1485.78	10^{-5}	18.63
		Gr(I)/SiO_2/Pym Si		2660.76	10^{-5}	33.37
		AgNPs/SiO_2/Pym Si		2161.29	10^{-7}	2.71×10^{3}
		AgNPs/Gr(I)/SiO_2/Pym Si		4439.01	10^{-8}	5.57×10^{4}
1573	aromatic C–C stretching	SiO_2/Flat Si	Raman	11796.87	10^{-3}	N.A.
		Gr(I)/SiO_2/Flat Si	SERS	879.99	10^{-5}	7.46
		Gr(I)/SiO_2/Pym Si		2330.05	10^{-5}	19.50
		AgNPs/SiO_2/Pym Si		1809.97	10^{-7}	1.53×10^{3}
		AgNPs/Gr(I)/SiO_2/Pym Si		4398.05	10^{-8}	3.73×10^{4}
1649	aromatic C–C stretching	SiO_2/Flat Si	Raman	15806.67	10^{-3}	N.A.
		Gr(I)/SiO_2/Flat Si	SERS	1993.86	10^{-5}	12.61
		Gr(I)/SiO_2/Pym Si		3705.85	10^{-5}	23.44
		AgNPs/SiO_2/Pym Si		2290.48	10^{-7}	1.45×10^{3}
		AgNPs/Gr(I)/SiO_2/Pym Si		4735.72	10^{-8}	3.00×10^{4}

References

S1. Dai, C.-Y.; Wang, W.-C.; Tseng, C.-A.; Ding, F.-C.; Chen, Y.-T.; Chen, C.-C. Spatial confinement approach using Ni to modulate local carbon supply for the growth of uniform transfer-free graphene monolayers. *J. Phys. Chem. C* **2020**, *124*, 23094-23105.

S2. Song, I.; Park, Y.; Cho, H.; Choi, H. C. Transfer-free, large-scale growth of high-quality graphene on insulating substrate by physical contact of copper foil. *Angew. Chem. Int. Ed.* **2018**, *57*, 15374-15378.

S3. Ding, F.-C.; Dai, C.-Y.; Yao, C.-L.; Lai, C. Y.; Chen, C.-C. Laminar flow-assisted metal etching for the preparation of high-quality transfer-free graphene. *Chem. Mater.* **2022**, *34*, 5471-5483.

S4. Schneider, C. A.; Rasband, W. S.; Eliceiri, K. W. NIH image to ImageJ: 25 years of image analysis. *Nat. Methods* **2012**, *9*, 671-675.

S5. Zhang, N.; Zhang, K.; Zou, M.; Maniyara, R. A.; Bowen, T. A.; Schrecengost, J. R.; Jain, A.; Zhou, D.; Dong, C.; Yu, Z.; Liu, H.; Giebink, N. C.; Robinson, J. A.; Hu, W.; Huang, S.; Terrones, M. Tuning the Fermi level of graphene by two-dimensional metals for Raman detection of molecules. *ACS Nano* **2024**, *18*, 8876-8884.

S6. Briggs, N.; Bersch, B.; Wang, Y.; Jiang, J.; Koch, R. J.; Nayir, N.; Wang, K.; Kolmer, M.; Ko, W.; De La Fuente Duran, A.; Subramanian, S.; Dong, C.; Shallenberger, J.; Fu, M.; Zou, Q.; Chuang, Y.-W.; Gai, Z.; Li, A.-P.; Bostwick, A.; Jozwiak, C.; Chang, C.-Z.; Rotenberg, E.; Zhu, J.; van Duin, A. C. T.; Crespi, V.; Robinson, J. A. Atomically thin half-van der Waals metals enabled by confinement heteroepitaxy. *Nat. Mater.* **2020**, *19*, 637-643.

S7. Giovannetti, G.; Khomyakov, P. A.; Brocks, G.; Karpan, V. M.; van den Brink, J.; Kelly, P. J. Doping graphene with metal contacts. *Phys. Rev. Lett.* **2008**, *101*, 026803.

S8. Shin, J. H.; Kim, S. H.; Kwon, S. S.; Park, W. I. Direct CVD growth of graphene on three-dimensionally-shaped dielectric substrates. *Carbon* **2018**, *129*, 785-789.

S9. Lee, E.; Kim, J.; An, T. K. Direct growth of CVD graphene on 3D-architectured substrates for highly stable tactile sensors. *Chin. J. Phys* **2020**, *67*, 569-575.

S10. Yang, J.; Tang, L.; Luo, W.; Shen, J.; Zhou, D.; Feng, S.; Wei, X.; Shi, H. Light trapping in conformal graphene/silicon nanoholes for high-performance photodetectors. *ACS Appl. Mater. Interfaces* **2019**, *11*, 30421-30429.

S11. Rehman, M. A.; Roy, S. B.; Gwak, D.; Akhtar, I.; Nasir, N.; Kumar, S.; Khan, M. F.; Heo, K.; Chun, S.-H.; Seo, Y. Solar cell based on vertical graphene nano hills directly grown on silicon. *Carbon* **2020**, *164*, 235-243.

S12. Jiao, T.; Liu, J.; Wei, D.; Feng, Y.; Song, X.; Shi, H.; Jia, S.; Sun, W.; Du, C. Composite transparent electrode of graphene nanowalls and silver nanowires on micropyramidal Si for high-efficiency Schottky junction solar cells. *ACS Appl. Mater. Interfaces* **2015**, *7*, 20179-20183.

S13. Tan, L.; Han, J.; Zuo, J.; Huang, K.; Chen, J.; Wang, C.; Lu, W.; Fu, L. Graphene conformal-coated ceramics in arbitrary shape targeting smart widgets. *Adv. Mater. Interfaces* **2017**, *4*, 1700467.

S14. Yen, W.-C.; Medina, H.; Hsu, C.-W.; Chueh, Y.-L. Conformal graphene coating on high-aspect ratio Si nanorod arrays by a vapor assisted method for field emitter. *RSC Adv.* **2014**, *4*, 27106-27111.

S15. Watanabe, H.; Hayazawa, N.; Inouye, Y.; Kawata, S. DFT vibrational calculations of rhodamine 6G adsorbed on silver: analysis of tip-enhanced Raman spectroscopy. *J. Phys. Chem. B* **2005**, *109*, 5012-5020.

S16. Arzumanyan, G.; Doroshkevich, N.; Mamatkulov, K.; Shashkov, S.; Girel, K.; Bandarenka, H.; Borisenko, V. Phospholipid detection by surface-enhanced Raman scattering using silvered porous silicon substrates. *Phys. Status Solidi A* **2017**, *214*, 1600915.
